# Structural analysis of the genome of breast cancer cell line ZR-75-30 identifies twelve expressed fusion genes

**DOI:** 10.1186/1471-2164-13-719

**Published:** 2012-12-22

**Authors:** Ina Schulte, Elizabeth M Batty, Jessica CM Pole, Katherine A Blood, Steven Mo, Susanna L Cooke, Charlotte Ng, Kevin L Howe, Suet-Feung Chin, James D Brenton, Carlos Caldas, Karen D Howarth, Paul AW Edwards

**Affiliations:** 1 Hutchison/MRC Research Centre and Department of Pathology, University of Cambridge, Cambridge, UK; 2 Cancer Research UK Cambridge Research Institute and Department of Oncology, University of Cambridge, Li Ka-Shing Centre, Cambridge, UK; 3 Current addresses: Department of Statistics, University of Oxford, 1 South Parks Road, Oxford, OX1 3TG, UK; 4 Current addresses: BlueGnome Ltd, CPC4, Capital Park, Fulbourn, Cambridge, CB21 5XE, UK; 5 Current addresses: Department of Medical Genetics, University of British Columbia, Vancouver, BC, V6H 2N1, Canada; 6 Current addresses: Institute of Biomedical Engineering, Department of Engineering Science, University of Oxford, Oxford, OX3 7DQ, UK; 7 Current addresses: Cancer Genome Project, Wellcome Trust Sanger Institute, Hinxton, Cambridgeshire, CB10 1SA, UK; 8 Current addresses: Breakthrough Breast Cancer Research Centre, Institute of Cancer Research, 237 Fulham Road, London, SW3 6JB, UK; 9 Current addresses: European Bioinformatics Institute, Hinxton, Cambridgeshire, CB10 1SD, UK

**Keywords:** Breast cancer, Chromosome aberrations, Genomics, Fusion genes

## Abstract

**Background:**

It has recently emerged that common epithelial cancers such as breast cancers have fusion genes like those in leukaemias. In a representative breast cancer cell line, ZR-75-30, we searched for fusion genes, by analysing genome rearrangements.

**Results:**

We first analysed rearrangements of the ZR-75-30 genome, to around 10kb resolution, by molecular cytogenetic approaches, combining array painting and array CGH. We then compared this map with genomic junctions determined by paired-end sequencing. Most of the breakpoints found by array painting and array CGH were identified in the paired end sequencing—55% of the unamplified breakpoints and 97% of the amplified breakpoints (as these are represented by more sequence reads). From this analysis we identified 9 expressed fusion genes: *APPBP2-PHF20L1, BCAS3-HOXB9, COL14A1-SKAP1, TAOK1-PCGF2, TIAM1-NRIP1, TIMM23-ARHGAP32, TRPS1-LASP1, USP32-CCDC49* and *ZMYM4-OPRD1*. We also determined the genomic junctions of a further three expressed fusion genes that had been described by others, *BCAS3-ERBB2, DDX5-DEPDC6/DEPTOR and PLEC1-ENPP2*. Of this total of 12 expressed fusion genes, 9 were in the coamplification. Due to the sensitivity of the technologies used, we estimate these 12 fusion genes to be around two-thirds of the true total. Many of the fusions seem likely to be driver mutations. For example, PHF20L1, BCAS3, TAOK1, PCGF2, and TRPS1 are fused in other breast cancers. *HOXB9* and *PHF20L1* are members of gene families that are fused in other neoplasms. Several of the other genes are relevant to cancer—in addition to ERBB2, SKAP1 is an adaptor for Src, DEPTOR regulates the mTOR pathway and NRIP1 is an estrogen-receptor coregulator.

**Conclusions:**

This is the first structural analysis of a breast cancer genome that combines classical molecular cytogenetic approaches with sequencing. Paired-end sequencing was able to detect almost all breakpoints, where there was adequate read depth. It supports the view that gene breakage and gene fusion are important classes of mutation in breast cancer, with a typical breast cancer expressing many fusion genes.

## Background

In the last few years it has emerged that the common epithelial cancers, such as carcinoma of breast, prostate and lung, have fusion genes like those long associated with leukaemias, lymphomas and sarcomas [1,2]. The first to be discovered were in prostate cancer, where about half of all cases have the TMPRSS2-ERG fusion gene [3,4], and lung cancer, where around 5% of lung cancers have a fusion that activates the ALK tyrosine kinase, the EML4-ALK fusion [5]. However, these early examples were found by essentially ‘one-off’ methods, and did not answer the question of how many fusions a typical carcinoma expresses ([4,5] reviewed in [1]).

In addition to creating fusion genes, the abundant genome rearrangements in these cancers break many other genes, and since breakage will almost always affect gene function, rearrangement is likely to make a significant contribution to inactivating genes [1,6].

Recent technical developments now allow systematic searches for genome rearrangements and hence fusion genes [1]. ‘Array painting’, i.e. hybridization of individual chromosomes to a genomic microarray, allows many chromosome rearrangements (though not inversions) to be analyzed to almost 1kb resolution [7-9]. ‘Paired-end-sequencing’ can be used to identify rearrangements by finding breakpoint junctions: small genomic DNA fragments, typically 250-500bp, are sequenced from both ends and the paired sequence reads examined to see whether they are the expected distance apart on the reference genome [10-12]. A variation is ‘mate-pairs’, where fragments of 3 to 5 kb are end-sequenced [11]. Paired-end sequencing is also being applied to cDNA to find fusion transcripts directly [13-15].

To search for fusion genes in a representative breast cancer we chose the ZR-75-30 breast cancer cell line [16]. It has a typically rearranged karyotype, and a typical high-copy-number coamplification of parts of chromosomes 8 and 17, particularly 8q24 and 17q11-24, forming five homogeneously staining regions (hsrs) [17]. As often seen in breast cancer [18-22], this is a complex coamplification of many small fragments of the genome. The amplification is relevant to the search for fusion genes as some amplifications harbour fusion genes, perhaps formed early in cancer development and subsequently amplified [10,20,21]. ZR-75-30 is also of interest as it is estrogen-receptor-positive (ER+) and has been used as a model of an ER+ breast cancer that is insensitive to tamoxifen, in contrast to the sensitive line ZR-75-1 (which was from an unrelated patient) [16].

To find fusion transcripts in ZR-75-30, we refined our previous 1-Mb resolution array-painting analysis of its karyotype [8], using high-resolution array CGH data. Then we applied paired-end sequencing to identify rearrangement junctions, particularly those in the amplification, which are preferentially sampled because they are present in multiple copies.

## Materials and methods

### Nomenclature, genome positions and transcripts

Genome positions are relative to GRCh37/hg19. Exon numbering is from the Ensembl transcripts listed in Additional file 1. Gene names follow HUGO Gene Nomenclature and protein reference numbers are from UniProtKB/Swiss-Prot database.

### Cells, DNA, RNA

ZR-75-30 cells were as used previously [8,17], derived from a sample frozen in 1999 by Dr M.J. O’Hare, Ludwig institute for Cancer Research/UCL Breast Cancer Laboratory, London, U.K., who had obtained them from the American Type Culture Collection. We authenticated them by STR (short tandem repeat) analysis, and they matched the ATCC database at all eight specified loci. Further evidence for their authenticity was that the fusion genes we described were common to other stocks of the line held by the ATCC and other laboratories (see Results). The cells were maintained on 50:50 DMEM:F12 medium (Invitrogen, Grand Island, NY, USA), 10 μg/ml insulin, 10% foetal bovine serum. Non-cancer breast cell lines, used to investigate expression in normal breast, were from the originators: HB4a is a line immortalized from purified breast luminal epithelial cells [23] and the HMT3522 line was from fibrocystic (non-cancer) breast [24]. Other breast cancer cell lines were as described [17,25]. Genomic DNA, total RNA and random-primed cDNA were prepared as described [26].

### Array-CGH data

Data were kindly provided by the Wellcome Trust Sanger Institute [27]. Breakpoint intervals were judged by eye and confirmed by segmentation using the PICNIC algorithm [28].

### Paired-end sequencing

ZR-75-30 genomic DNA was sequenced in paired-end read mode using the Illumina GAIIx Genome Analyzer, and HiSeq2000 (Illumina, Great Chesterford, UK) [10,29]. Briefly, we sheared 5 μg of genomic DNA by sonication using a Bioruptor sonicator (Diagenode, Liège, Belgium). The fragmented DNA was end-repaired and a 3’ overhang was created, followed by ligation of Illumina paired-end adaptor oligonucleotides. We size-selected fragments at 400–600 bp by agarose gel electrophoresis, and enriched for fragments with primers on either end by an 18-cycle PCR reaction. A total of five flowcell lanes were sequenced. 43 million, 36-bp, paired sequences (counting only unique reads with high-quality mapping) were obtained from one 500 bp library (median 504 bp, range 404 – 619 bp), equivalent to average 1.7-fold coverage of single-copy breakpoints in this subtetraploid genome.

Two additional paired-end sequencing libraries were made by the ‘mate-pair’ approach [11]: 3kb DNA fragments were circularized and the junction fragments isolated as a paired-end library, using reagent kits supplied by Illumina. A single lane of each 3 kb library was sequenced, yielding about 1.25 million paired sequences, equivalent to 0.5 X coverage of single-copy breaks.

### Alignment and fusion prediction

In outline, analysis steps were: (i) alignment of sequencing reads, (ii) identifying aberrant pairs of read pairs, i.e. read pairs that aligned but not in the expected orientation or separation, (iii) clustering concordant aberrant reads to find candidate structural variants, and filtering of those candidates, (iv) prediction and verification of fusion genes.

Raw sequences were obtained from Illumina’s standard image analysis (FIRECREST) and base calling modules (BUSTARD). Reads were aligned to the reference genome GRCh37/hg19 with BWA [30] to identify and remove normal read pairs, which align to the genome with the expected distance apart and orientation. Non-normal reads were then realigned using Novoalign (Novocraft Technologies, Selangor, Malaysia), a slower but more thorough aligner. Novoalign gives each read a mapping quality score, a measure of the confidence of mapping, and read pairs in which either read scored below 30 were discarded. Library preparation involved a PCR amplification step which can result in duplicate copies of the same read pair being sequenced: exact PCR duplicates were identified, and all but one copy removed, using Picard (http://picard.sourceforge.net/;[31]). This gave ’aberrant read pairs’, read pairs that aligned but not with normal separation and orientation. These were then grouped into clusters of read pairs that were consistent with the same rearrangement junction: a minimum of two consistent reads were required. Additional filters were then applied. Read pairs were checked for a possible normal match to the reference genome using BLAT [32], since the alignment software sometimes aligns a read to an homologous sequence instead of its true match, perhaps because of sequencing errors or polymorphisms. Likely PCR duplicates that were offset by one or two bp were also discarded as likely to be PCR duplicates where a primer had lost one or two 3’ base pairs. Known normal human copy number variations [33] were discarded. Apparent variants were removed if they also appeared in a pool of paired end sequences from 18 other unrelated samples from cancers, normal tissue or cell lines. Apparent intra-chromosomal rearrangements spanning less than 10kb were also discarded, as most would be polymorphisms or outsize fragments. (Note that this does not remove all small rearrangements, such as small apparent insertions, e.g. the two apparent junctions between chromosome 1 at 109.65 Mb and a fragment of chr22 at 30.16Mb. Such ‘insertions’ may be deletions in the reference genome).

Gene fusions and breakage were predicted from the resulting rearrangement breakpoints using the Ensembl Application Programming Interface http://www.ensembl.org/info/docs/api/index.html to retrieve all the genes that overlapped the breakpoints, or were adjacent to breakpoints. To predict whether a fusion transcript could be formed we considered whether the 5’ or 3’ end of a gene would be retained, and whether, when the 5’ end of a gene was retained, a ‘runthrough’ fusion could be formed by transcription into a downstream intact gene near the junction.

### Verification, Cloning and Sequencing of Junctions

Selected genomic junctions were verified by PCR using primers designed to flank the junction (Additional file 2 and Additional file 3; Eurofins MWG Operon, Ebersberg, Germany), using DNA pooled from twenty normal individuals as a control. To detect fusion transcripts, we amplified from cDNA using primers in flanking exons of the expected fusions. Selected full-length transcripts were then amplified using primers designed to include the putative start and stop codons. Amplification was for 35 cycles with an annealing temperature of 58°C using HotMaster Taq DNA Polymerase (5 PRIME GmbH, Hamburg, Germany) or, for long-range PCR, Elongase® Enzyme Mix (Invitrogen, Carlsbad, CA, USA) with 2mM Mg^2+^. PCR products were sequenced in both directions, generally after cloning using a TOPO TA cloning kit (Invitrogen, Carlsbad, CA, USA). Primers used for cloning genomic and cDNA junctions are given in Additional file 4.

This study did not require ethical approval.

## Results

### Refined cytogenetic map of the ZR-75-30 genome

We first refined our previous analysis of the karyotype of ZR-75-30 to ~10kb resolution. In our previous analysis we used array painting, in which chromosomes are isolated by flow cytometry and hybridized individually to genomic arrays, to identify the components of each chromosome [8]. This had given us a map of inter-chromosome rearrangements spanning more than about 3 Mb. This analysis was refined by matching the unbalanced breakpoints with array-comparative genomic hybridization (array-CGH) on the SNP6 platform, from Bignell et al. [27] (Additional file 5). Some additional copy number steps, below the resolution of the array painting, were revealed in the unamplified regions, notably additional breaks on chromosome 1 (which are most likely additional internal rearrangement of the 1;21 chromosome translocation named peak G in Howarth et al. [8]) (Additional file 5 and Additional file 6).

We then overlaid a list of breakpoint junctions obtained by paired-end sequencing (Additional file 5). These junctions had been filtered in various ways to reduce artefacts (see Methods). We additionally required junctions to be identified by at least two independent read pairs in one library and either (i) to be present in more than one of the three libraries sequenced or (ii) to correspond to a copy number step, in the SNP6 array-CGH data [27] or the array painting data [8].

**Figure 1:**
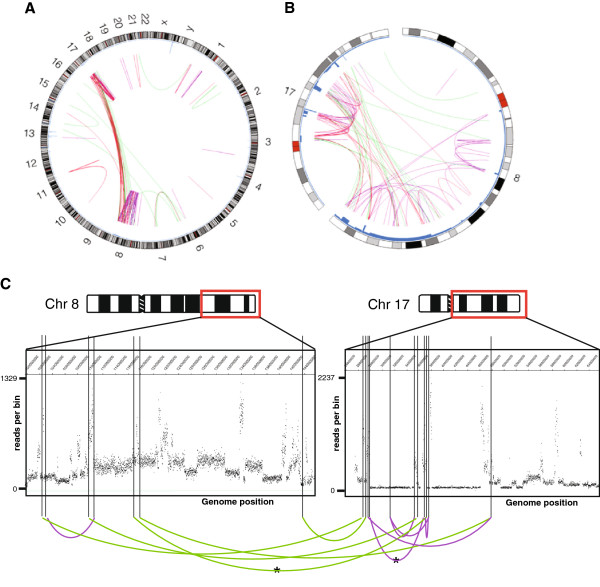
**Chromosome rearrangements observed in the genome of breast cancer cell line ZR-75-30. A**. Genome-wide Circos plot of structural variation in the ZR-75-30 genome. An ideogram of a normal karyotype is shown around the outside. Copy number variation is represented by the blue line, shown inside the ideogram. Chromosome rearrangements are depicted with green (interchromosomal) and purple (intrachromosomal) lines. All 125 structural variants shown have been independently validated by PCR (red lines) or by matching to a copy number step on a SNP6 array. **B**. Structural variation in the complex 8;17 amplicon. Colours as in A. **C**. Copy number variation in the 8;17 amplicon of ZR-75-30. Ideograms of chromosomes 8 and 17 are shown, with regions containing amplified segments highlighted with a red box and expanded below. Copy number changes, as measured by paired-end sequencing, illustrate the complexity of the amplification. An example set of ten confirmed rearrangement junctions are shown with green (interchromosomal) and purple (intrachromosomal) lines. Genome positions are based on Hg19.

This strategy yielded 318 apparent genomic junctions (Additional file 7), of which 112 were identified as likely to explain a copy number step or match a junction in the array painting data (Additional file 5). Of the 318 genomic junctions, we identified 47 that were predicted to fuse genes, and tested for them by PCR on genomic DNA. 37/47 junctions were successfully amplified, among which 24/25 junctions were amplified that were associated with copy number steps, compared to 13/22 that were not. 2 of these 13 junctions, not associated with detectable copy number change, were also amplified from pooled normal genomic DNAs and therefore were not considered further. The 125 genomic junctions that had been confirmed by an associated copy number step (89), or positive PCR product (13), or both (23), are illustrated in Figure 1 and Additional file 2. 62% of these are intra-chromosomal rearrangements.

We were able to identify breakpoint junctions corresponding to most of the previously-known breakpoints: about 55% of the breakpoints in unamplified regions, and 97% of the breakpoints (identified from copy number steps) in the amplified regions of chromosome 8 and 17, which, because they are present in many copies, gave more reads in the sequencing (Additional file 5 and Additional file 7 and Figure 1).

The array-CGH showed that the coamplification of chromosomes 8 and 17 was very complex (Figure 1C), too complex for all the fragments and copy number steps to be resolved. A reliable map of the amplicon cannot be assembled from these junctions alone, because not all junctions would have been detected, some may be spurious, and there are usually multiple ways to assemble a given set of junctions into a linear map [34,35]. However, we show one possible assembly of 10 of the junctions from chromosomes 8 and 17, to illustrate the complexity (Additional file 8). There was also a junction, verified by genomic PCR, that may well represent the join between the 8;17 amplification and flanking chromosome 14 material. It joins 84.97 Mb on chromosome 14 to 102.54 Mb on chromosome 8. All four chromosomes that carry blocks of 8;17 coamplification also carry 14q (chromosome fractions C,D, F and L in ref. [8]), so this join may be the same on all of them.

### Gene fusions

We found a total of 12 expressed gene fusions: we predicted 9 from paired-end sequencing, and we confirmed a further 3 that were reported by Robinson DR et al. [15], also identifying the structural rearrangements that had generated these additional fusions.

**Figure 2:**
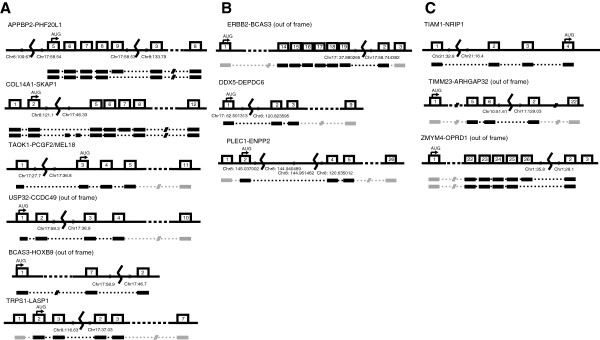
**Schematic representation of gene fusions and the expressed fusion transcripts in the breast cancer cell line ZR75-30 (not to scale). A**. Fusions in the 8;17 amplicon. **B**. Structure of fusion transcripts detected by Robinson et al. [15]. **C**. Fusions at single copy breaks. Relevant exons are represented as numbered boxes, the transcription start site (AUG) is indicated with a black arrow and the breakpoint is indicated with a zig-zag line at the approximate chromosomal position (based on the UCSC Genome Browser, hg19). The (sequenced) expressed fusion transcripts and (where applicable) alternative splice products are shown below as black boxes joined by a dotted line. Exons depicted in grey are expected to be expressed, but were not sequenced. For numbering of exons see Additional file 1.

Our nine fusion genes were found by searching junctions computationally to identify potentially fused genes, followed by manual inspection (Additional file 7). Junctions predicted to create fusions were verified by PCR on genomic DNA, as above, and the predicted transcripts were tested for by PCR from cDNA. Of thirty predicted fusion transcripts, nine were successfully amplified (Table 1, Figure 2; for junction sequences see Additional file 1 and Additional file 3), including two of thirteen predicted ‘run-through’ fusions, i.e. fusions formed by breakage of the 5’ gene and transcription from this gene into an intact downstream gene (Figure 2). Some of the failures to amplify junctions and fusion transcripts may have been technical failures, or due to errors in mapping the paired sequences, or because the rearrangements were more complex than the automated analysis revealed.

**Table 1:** Verified expressed gene fusions in the breast cancer cell line ZR-75-30 predicted from structural analysis

**5’ gene**	**3’ gene**	**Chromosomes involved**^**a**^		**Expression**	**In frame**^**e**^	**Fusion (F) or Runthrough fusion (R)**
		**5’**	**3’**			
APPBP2	PHF20L1	17	8	yes	yes	F
COL14A1	SKAP1	8	17	yes	yes	F
TAOK1	PCGF2	17	17	yes ^b,d^	yes	F
USP32	CCDC49	17	17	yes ^d^	no	F
BCAS3	HOXB9	17	17	yes ^d^	see text	F
TRPS1	LASP1	8	17	yes	yes	R
ERBB2	BCAS3	17	17	yes ^c^	no	R
DDX5	DEPDC	17	8	yes ^c^	yes	R
PLEC1	ENPP2	8	8	yes ^c^	yes	F
TIAM1	NRIP1	21	21	yes ^b^	yes	F
ZMYM4	OPRD1	1	1	yes	no	F
TIMM23	ARHGAP32	10	11	yes	no	R
TMEM74	APPBP2	8	17	no		F
TRAPPC9	STARD3	8	17	no		F
SSH2	PLXDC1	17	17	no		F
TAOK1	CA10	17	17	no		F
HYLS1	TIMM23	11	10	no ^b^		F
USP32	RALYL	17	8	no		F
TMEM74	ACACA	8	17	no		F
NUDCD1	TAC4	8	17	no		R
TRAPPC9	HOXB6	8	17	no		R
SSH2	NFE2L1	17	17	no		R
TTC35	MKS1	8	17	no		R
TMEM71	CRYBA1	8	17	no		R
CA3	KIAA1429	8	8	no		R
GRHL2	NUDCD1	8	8	no		R
SUPT6H	GPIHBP1	8	17	no		R
PGAP3	NOV	8	17	no		R
KIAA0100	LY6H	8	17	no		R
TG	ERBB2	8	17	no		R

All genomic junctions tested were positive by PCR; those marked c were not tested.

^a^ Precise chromosomal positions are given in Additional file 2 and Additional file 5 and the exon structure in Figure 2.

^b^ 5’ gene is untranslated sequence only.

^c^ Fusions not predicted by our analysis but detected by transcriptome sequencing by Robinson et al. (2011) and confirmed here by RT-PCR. Genomic breakpoints were detected in the present dataset on additional inspection—they had not met our stringent criteria or were complex rearrangements.

^d^ Fusions also reported by Robinson et al. (2011).

^e^ Predicted from annotations; not experimentally verified.

We showed, by PCR, that all twelve of the fusions were present in other available stocks of the ZR-75-30 cell line, and not recent evolution in our cultures. All the fusion transcripts were present in the ZR-75-30 stock used in Robinson et al. [15], tested using RNA kindly provided for the purpose by Prof Reis-Filho, Breakthrough Breast Cancer Research Centre, Institute of Cancer Research, London, UK (passage 5 after receipt from ATCC), and in a separate stock from the Institute of Cancer Research. Furthermore, the genomic junctions that create the twelve fusions were all present in a DNA sample newly purchased direct from ATCC.

As found previously in breast cancer cell lines [12,36], a number of the genomic breakpoint junctions showed microhomology (four out of seven sequenced junctions had 1–4 bp of microhomology), and one contained a small fragment of sequence inserted from elsewhere in the genome, termed a ‘genomic shard’ [37] (Additional file 3). This may be characteristic of a microhomology-mediated break-induced-replication (MMBIR) mechanism [38]. Our strategy may overlook some of these complex junctions.

Of the 12 fusions (Figure 2), nine were from the coamplification of chromosomes 8 and 17. Four were ‘run-through fusions’, where transcription runs from a broken 5’ gene into an intact downstream gene, with splicing into the first splice acceptor, usually the second exon. Two fusions spanned two or more junctions (Figure 2A and B).

### Fusion genes in the (8;17) amplicon

#### APPBP2-PHF20L1

Paired-end reads suggested a complex rearrangement that joined part of *APPBP2* and *PHF20L1* (Figure 2A; Additional file 7). We confirmed the presence of a double junction at the genomic level by amplifying the expected 10.4 kb *APPBP2* insert by long-range PCR between chromosome 8 and *PHF20L1*-intron 2 (Additional file 3).

A fusion transcript was detected that splices exon 9 of *APPBP2* in frame to exon 3 of *PHF20L1* Isoform 2 (ENST00000337920: the ENSEMBL transcripts from which the exon numbering was taken are listed in Additional file 1) (Figure 2A). Additionally, an alternatively spliced, out-of-frame fusion transcript was detected (Figure 2A).

This is likely to be only part of the fusion transcript, since exon 5 is not a known transcription start site of APPBP2. The upstream genomic junction (Figure 2A) joins *APPBP2* intron 4 to chromosome 8 at 109.67 Mb, but does not join it to any known gene—presumably there is a further rearrangement junction upstream of this.

#### COL14A1-SKAP1

A full-length fusion transcript was amplified in which *COL14A1* exon 2 was joined in frame to exon 5 of *SKAP1* (Table 1, Figure 2A, Additional file 1 and Additional file 7). Additional products were amplified that included cryptic exons of varying length from within intron 4 of *SKAP1* (Figure 2A), but in these transcripts *SKAP1* is out of frame. Additionally, a splice variant lacking exon 7 was found, introducing a stop codon in exon 8. Exon-7-skipped transcripts were observed in other breast cancer cell lines. It is not clear whether SKAP1 is upregulated by fusion as its expression was very variable among normal and breast cancer cell lines. In T lymphocytes SKAP1 is associated with ADAP, and ADAP mRNA was detected in ZR-75-30 and other cell lines with relatively high SKAP1 expression.

#### TAOK1-PCGF2/MEL18, USP32-CCDC49, BCAS3-HOXB9, TRPS1-LASP1

The *TAOK1-PCGF2, USP32-CCDC49, and BCAS3-HOXB9* fusion transcripts were detected by RT-PCR essentially as expected (Figure 2A), except that the splice donor and acceptor sites of *TAOK1* and *PCGF2* in their fusion transcript were both offset a few base pairs from the splice junctions reported by ENSEMBL (Additional file 1). These three fusions were also detected by transcriptome sequencing [15]. The *TRPS1-LASP1* fusion joins exon 3 of the transcription factor *TRPS1*, by transcription running through, in frame, to exon 2 of *LASP1* (Figure 2A).

#### BCAS3-ERBB2, DDX5-DEPDC6/DEPTOR, PLEC1-ENPP2

These three fusion transcripts were not discovered by our initial analysis. They were reported by Robinson et al. [15], who detected 6 fusion transcripts in ZR-75-30 by sequencing cDNA, three of which we had found (Table 1). The additional three fusion transcripts we confirmed, by RT-PCR, and we also identified their genomic junctions in our sequencing data (Figure 2B). We had failed to discover two of these fusions because of limitations of our fusion prediction: *DDX5-DEPDC6/DEPTOR* is a ‘run-through’ fusion (see above) that had been obscured by other possible downstream fusion partners; while *PLEC1-ENPP2* was formed by a complex rearrangement apparently comprising two genomic junctions (Figure 2B). The *BCAS3-ERBB2* breakpoint junction was present only in one mate-pair library and therefore had not met our stringent criteria.

### Fusion genes not in the amplicon, *TIAM1-NRIP1, TIMM23-ARHGAP32*, *ZMYM4-OPRD1*

The *TIAM1-NRIP1* fusion was predicted both from paired-end sequencing and from combining the array painting with SNP6 array-CGH. It was probably formed by a simple 16-Mb interstitial deletion on chromosome 21, since the presumed deleted region is at lower copy number in array CGH and absent from the array painting hybridisation of chromosome der(1)t(1;21)del(21) (peak G in [8]). A full-length transcript was amplified, with *TIAM1* exon 1 fused to *NRIP1* exon 2 (Table 1, Figure 2C, Additional file 1).

The *TIMM23-ARHGAP32* fusion is the result of a translocation between chromosomes 10 and 11. *TIMM23* is broken and transcription runs into the intact *ARHGAP32* gene, joining exon 6 of *TIMM23* to exon 2 of *ARHGAP32* (Figure 2C).

The *ZMYM4-OPRD1* fusion is the result of an internal rearrangement of chromosome 1 (Table 1, Figure 2C, Additional file 7). Two transcripts were observed, both joining *OPRD1* out of frame and leading to a stop codon shortly after the breakpoint. A major transcript was detected, fusing *ZMYM4*-exon 26 to *OPRD1*-exon 2 as expected (Figure 2C), and a minor transcript, splicing *ZMYM4*-exon 25 to *OPRD1*-exon 2 (Figure 2C).

We were unable to clone and sequence the *ZMYM4-OPRD1* genomic junction, but several junctions were detected in this region of chromosome 1, suggesting that the rearrangement may be complex.

## Discussion

### Analysis of the ZR-75-30 genome

Together, these data provide a gene-level analysis of most of the unamplified genome rearrangements in this cell line, of more than 10 kb span. A few details are still missing, notably the centromeric breakpoints, and some balanced breakpoints. Balanced breakpoints are invisible to array-CGH and not all were sampled by the paired-end sequencing or fine-mapped in our previous array painting.

Paired-end sequencing has various limitations, and combining with other structural data as we have done is clearly valuable. Firstly, the method is not expected to find all rearrangements, because it samples the genome at random, and coverage is dependent on GC content [29]. Also, reads in repeats and segmental duplications generally cannot be used because they cannot be mapped to a unique match in the reference genome. Secondly, artefactual rearrangements can be created by coligation of DNA fragments during preparation for sequencing, and by errors in mapping reads.

Sampling of junctions was surprisingly good: we accounted for 97% of the copy number steps detected by array-CGH in the amplicon, where the greater number of reads across the junctions increased sensitivity. This suggests that, even using only 36 bp reads, rather few junctions would be undetectable because they are flanked by non-unique sequences. The lower sampling of single-copy junctions resulted in about 55% of the junctions detected by array-CGH being detected by sequencing. Conversely, we identified almost twice as many junctions in the amplicon as we expected from the copy number steps. These were presumably a mixture of artefacts and additional rearrangements that are not resolved by CGH, either because they involve small fragments or are balanced.

Another limitation of paired end sequencing is that it does not show how junctions are joined together, e.g. whether two apparently-neighbouring junctions are on the same chromosome or not, nor whether the region between is interrupted by further junctions [35]. This is illustrated by two of the fusion genes, *APPBP2-PHF20L1* and *PLEC1-ENPP2,* both transcribed across more than one genomic junction.

### ZR-75-30 expresses at least 12 fusion transcripts

By combining molecular cytogenetic approaches—high-resolution array-CGH and array painting—with paired-end sequencing, we have catalogued genome rearrangements of this cell line and found 9 expressed fusion transcripts. We combined this with 3 additional fusion transcripts found by sequencing cDNA [15], for which we have identified the genomic junctions.

Nine of 12 fusions in ZR-75-30 are in the complex coamplification of chromosomes 8 and 17, the fusions *APPBP2-PHF20L1, BCAS3-HOXB9, TAOK1-PCGF2* and *DDX5-DEPDC6/DEPTOR* being most amplified. Such complex coamplifications are common [19] and probably give the ‘firestorm’ pattern of multiple small amplified fragments seen in array-CGH [22,39]. The MCF7 cell line has a similar coamplification involving chromosomes 1, 3, 17, and 20 and containing highly-amplified gene fusions [6].

Of these 12 fusion genes, seven were formed by intra-chromosomal rearrangements, confirming that more fusion genes are formed by intra-chromosomal rearrangement than by chromosome translocation [1]. This might be expected if rearrangements arise at replication bubbles [36] rather than random breakage and rejoining.

### How many expressed fusion genes are there in breast cancers?

Extrapolating from our work and Robinson DR et al. [15], ZR-75-30 may have around 18 expressed fusion genes and breast cancers in general—not cell lines—may express on average around 10.

In ZR-75-30, using structural analysis, we found half of the six expressed fusions detected by Robinson DR et al. [15], while, using cDNA sequencing, they found three of the nine we detected—both figures suggest the true total might be around 18. This is consistent with recent, probably incomplete, figures from other cell lines: 20 expressed fusions have been verified in MCF7, with several more predicted computationally [6,13,15,40]; 43 have been found in BT474 and 13 in SKBR3 [13].

Breast cancers—as opposed to cell lines—appear to have almost as many fusions. Robinson DR et al. [15] identified an average of 4.2 expressed fusions per case (0 to 20 in 38 breast tumours), compared to 5.5 per case in cell lines. Their sensitivity seems to have been around 40%, comparing their findings with ours and with the published cell line data above. This gives a best guess that breast tumours will on average express 10 fusions [41], with wide variation from cases to case, as expected from their variable levels of rearrangement [42].

### Are these passenger or driver mutations?

The fusions found here argue strongly that some at least are selected, i.e. ‘driver’ mutations, rather than random incidental ‘passenger’ mutations [43]. As detailed in the supplementary discussion in Additional file 9, several of the genes involved have already been found to be fused in other breast cancer cell lines—*PHF20L1 and BCAS3*[6,13,15,21,44] —or in other tumours—*BCAS3* again, and *PCGF2, TAOK1 and TRPS1*[45,46]. Others are members of families that include multiple fused genes—the collagens, *HOX* and *PHF* families. Several of the fusions resemble known recurrent gene fusions in general functional terms [1,2]: for example, fusions of *HOXB9, PCGF2, PHF20L1*, and *NRIP1* would be typical of the many known fusions that control gene expression directly or via chromatin structure, and all could encode functional domains of the proteins. Several of the genes involved are also in signalling pathways relevant to breast cancer: ERBB2, NRIP1 and BCAS3 are involved in estrogen receptor function and APPBP2 with androgen receptor; while TAOK1 and SKAP1 are involved in MAPK signalling and DEPDC6/DEPTOR regulates mTOR signalling.

Several of the fused genes are also recurrently broken in a substantial proportion of breast cancers, as judged by copy number steps in array-CGH of 1000 breast tumours [47]: around 10% have breaks in ERBB2, BCAS3 and SKAP1, while COL14A1, TIAM1, USP32, TAOK1 are broken in around 4%.

Some of the fusions, and particularly those not expressed, may simply inactivate a copy of the participating gene(s) [1,6]. For example, our fusions of *TIAM1* and *TAOK1* inactivate one copy of these genes. Some genes, e.g. *BCAS3*, that are fused in more than one cancer cell line retain different, non-overlapping parts of the gene in different cases, suggesting the common theme is inactivation. In some cases fusion of a gene may suppress its expression, perhaps by destabilising the mRNA: among the predicted fusion genes for which we could not detect a transcript, unfused copies of some of the 5’ participating genes were transcribed—for example *SSH2, NUDCD1* and *TRAPPC9* (Table 1; Additional file 7).

## Conclusion

### Fusion genes in ZR-75-30 and cancers in general

We have brought the total of fusion genes expressed by ZR-75-30 to 12, and there are good reasons to think the final total will be around 18. We have argued from this and other data that carcinomas not only have fusion genes analogous to those found in leukaemias [1,4], but each case may have many of them, and many will be functionally significant. This suggests a picture of neoplasia in which all neoplasms have a mixture of mutation types—point mutations, deletions, fusion genes, etc. Rather than leukaemias being driven by fusion genes while carcinomas were driven by point mutations and deletions, the main difference between carcinomas and leukaemias may simply be that carcinomas have more mutations than leukaemias.

## Abbreviations

*APPBP2*: Amyloid beta precursor protein (cytoplasmic tail) binding protein 2; *PHF20L1*: PHD finger protein 20-like 1; *BCAS3*: Breast carcinoma amplified sequence 3; *HOXB9*: Homeobox B9; *COL14A1*: Collagen, type XIV, alpha 1; *SKAP1*: src kinase associated phosphoprotein 1; *TAOK1*: TAO kinase 1; *PCGF2*: Polycomb group ring finger 2; *TIAM1*: T-cell lymphoma invasion and metastasis 1; *NRIP1* (RIP140): Nuclear receptor interacting protein 1; *TIMM23*: Translocase of inner mitochondrial membrane 23 homolog (yeast); *ARHGAP32*: Rho GTPase activating protein 32; *TRPS1*: Trichorhinophalangeal syndrome I; *LASP1*: LIM and SH3 protein 1; *USP32*: Ubiquitin specific peptidase 32; *CCDC49*: (CWC25) spliceosome-associated protein homolog (S. cerevisiae); *ZMYM4*: Zinc finger MYM-type protein 4; *OPRD1*: Opioid receptor, delta 1; *ERBB2*: v-erb-b2 erythroblastic leukemia viral oncogene homolog 2; *DDX5*: DEAD (Asp-Glu-Ala-Asp) box polypeptide 5; *DEPDC6/DEPTOR*: DEP domain containing MTOR-interacting protein; *PLEC1*: Plectin; *ENPP2*: Ectonucleotide pyrophosphatase/phosphodiesterase 2; *TMPRSS2*: Transmembrane protease, serine 2; *ERG*: v-ets erythroblastosis virus E26 oncogene homolog (avian); *ALK*: Anaplastic lymphoma receptor tyrosine kinase; *EML4*: Echinoderm microtubule associated protein like 4; ER+: Estrogen-receptor positive; Array-CGH: Array-comparative genomic hybridization; MAPK: Mitogen-activated protein kinase; *SSH2*: Slingshot homolog 2; *NUDCD1*: NudC domain containing 1; *TRAPPC9*: Trafficking protein particle complex 9.

## Competing interests

The authors declare that they have no competing interests.

## Authors’ contributions

IS, KDH, CC and PAWE conceived the study. IS, KDH, JCMP, KAB, SM and SFC carried out experiments. EMB and PAWE, with KDH, SLC, CN, KH and JDB analysed the sequencing data. IS, KDH and PAWE wrote the manuscript. All authors read and approved the final manuscript.

## Supplementary Material

Additional file 1 Junction and fusion transcript sequences. Click here for file

Additional file 2 Confirmed structural variants in ZR-75-30. Click here for file

Additional file 3 Genomic junction sequences. Click here for file

Additional file 4 Primers for amplifying genomic or transcript junctions and full-length fusion genes. Click here for file

Additional file 5 A comparison of breakpoints determined by snp6 and solexa sequencing. Click here for file

Additional file 6 A comparison of breakpoints by 1Mb array painting and solexa sequencing data. Click here for file

Additional file 7 Structural rearrangements determined by paired-end sequencing. Click here for file

Additional file 8 One possible assembly of ten junctions in the 8;17 amplicon of ZR-75-30. Click here for file

Additional file 9 Supplementary discussion: Discussion of individual fusion genes. Click here for file

## References

[B1] Edwards PAW Fusion genes and chromosome translocations in the common epithelial cancers J Pathol 2010 220 244 254 19921709 10.1002/path.2632

[B2] Mitelman F Johansson B Mertens F The impact of translocations and gene fusions on cancer causation Nat Rev Cancer 2007 7 233 245 10.1038/nrc2091 17361217

[B3] Mehra R Tomlins SA Shen R Nadeem O Wang L Wei JT Pienta KJ Ghosh D Rubin MA Chinnaiyan AM Shah RB Comprehensive assessment of TMPRSS2 and ETS family gene aberrations in clinically localized prostate cancer Modern pathology: an official journal of the United States and Canadian Academy of Pathology, Inc 2007 20 538 544 10.1038/modpathol.3800769 17334343

[B4] Tomlins SA Rhodes DR Perner S Dhanasekaran SM Mehra R Sun X-W Varambally S Cao X Tchinda J Kuefer R Recurrent fusion of TMPRSS2 and ETS transcription factor genes in prostate cancer Science (New York, NY) 2005 310 644 648 10.1126/science.1117679 16254181

[B5] Soda M Choi YL Enomoto M Takada S Yamashita Y Ishikawa S Fujiwara S-i Watanabe H Kurashina K Hatanaka H dentification of the transforming EML4-ALK fusion gene in non-small-cell lung cancer Nature 2007 448 561 566 10.1038/nature05945 17625570

[B6] Hampton OA Den Hollander P Miller CA Delgado DA Li J Coarfa C Harris RA Richards S Scherer SE Muzny DM A sequence-level map of chromosomal breakpoints in the MCF-7 breast cancer cell line yields insights into the evolution of a cancer genome Genome Res 2009 19 167 177 19056696 10.1101/gr.080259.108 PMC2652200

[B7] Fiegler H Gribble SM Burford DC Carr P Prigmore E Porter KM Clegg S Crolla JA Dennis NR Jacobs P Carter NP Array painting: a method for the rapid analysis of aberrant chromosomes using DNA microarrays J Med Genet 2003 40 664 670 10.1136/jmg.40.9.664 12960211 PMC1735585

[B8] Howarth KD Blood KA Ng BL Beavis JC Chua Y Cooke SL Raby S Ichimura K Collins VP Carter NP Edwards PAW Array painting reveals a high frequency of balanced translocations in breast cancer cell lines that break in cancer-relevant genes Oncogene 2008 27 3345 3359 10.1038/sj.onc.1210993 18084325 PMC2423006

[B9] Veltman JA Fridlyand J Pejavar S Olshen AB Korkola JE DeVries S Carroll P Kuo W-L Pinkel D Albertson D Array-based comparative genomic hybridization for genome-wide screening of DNA copy number in bladder tumors Cancer Res 2003 63 2872 2880 12782593

[B10] Campbell PJ Stephens PJ Pleasance ED O'Meara S Li H Santarius T Stebbings LA Leroy C Edkins S Hardy C Identification of somatically acquired rearrangements in cancer using genome-wide massively parallel paired-end sequencing Nat Genet 2008 40 722 729 10.1038/ng.128 18438408 PMC2705838

[B11] Korbel JO Urban AE Affourtit JP Godwin B Grubert F Simons JF Kim PM Palejev D Carriero NJ Du L Paired-end mapping reveals extensive structural variation in the human genome Science (New York, NY) 2007 318 420 426 10.1126/science.1149504 PMC2674581 17901297

[B12] Stephens PJ McBride DJ Lin M-L Varela I Pleasance ED Simpson JT Stebbings LA Leroy C Edkins S Mudie LJ Complex landscapes of somatic rearrangement in human breast cancer genomes Nature 2009 462 1005 1010 10.1038/nature08645 20033038 PMC3398135

[B13] Kim D Salzberg SL TopHat-Fusion: an algorithm for discovery of novel fusion transcripts Genome Biol 2011 12 R72 10.1186/gb-2011-12-8-r72 21835007 PMC3245612

[B14] McPherson A Hormozdiari F Zayed A Giuliany R Ha G Sun MGF Griffith M Heravi Moussavi A Senz J Melnyk N DeFuse: an algorithm for gene fusion discovery in tumor RNA-Seq data PLoS Comput Biol 2011 7 e1001138 10.1371/journal.pcbi.1001138 21625565 PMC3098195

[B15] Robinson DR Kalyana-Sundaram S Wu Y-M Shankar S Cao X Ateeq B Asangani IA Iyer M Maher CA Grasso CS Functionally recurrent rearrangements of the MAST kinase and Notch gene families in breast cancer Nature medicine 2011 17 1646 1651 10.1038/nm.2580 PMC3233654 22101766

[B16] Engel LW Young NA Tralka TS Lippman ME O'Brien SJ Joyce MJ Establishment and characterization of three new continuous cell lines derived from human breast carcinomas Cancer research 1978 38 3352 3364 688225

[B17] Davidson JM Gorringe KL Chin SF Orsetti B Besret C Courtay-Cahen C Roberts I Theillet C Caldas C Edwards PA Molecular cytogenetic analysis of breast cancer cell lines British journal of cancer 2000 83 1309 1317 10.1054/bjoc.2000.1458 11044355 PMC2408781

[B18] Guan X-Y Meltzer PS, Dalton WS, Trent JM: Identification of cryptic sites of DNA sequence amplification in human breast cancer by chromosome microdissection Nature genetics 1994 8 155 161 10.1038/ng1094-155 7842014

[B19] Paterson AL Pole JCM Blood KA Garcia MJ Cooke SL Teschendorff AE Wang Y Chin S-F Ylstra B Caldas C Edwards PAW Co-amplification of 8p12 and 11q13 in breast cancers is not the result of a single genomic event Genes, chromosomes & cancer 2007 46 427 439 10.1002/gcc.20424 17285574

[B20] Volik S Zhao S Chin K Brebner JH Herndon DR Tao Q Kowbel D Huang G Lapuk A Kuo W-L End-sequence profiling: sequence-based analysis of aberrant genomes Proceedings of the National Academy of Sciences of the United States of America 2003 100 7696 7701 10.1073/pnas.1232418100 12788976 PMC164650

[B21] Bärlund M Monni O Weaver JD Kauraniemi P Sauter G Heiskanen M Kallioniemi O-P Kallioniemi A Cloning of BCAS3 (17q23) and BCAS4 (20q13) genes that undergo amplification, overexpression, and fusion in breast cancer Genes, chromosomes & cancer 2002 35 311 317 10.1002/gcc.10121 12378525

[B22] Russnes HG Vollan HK Lingjaerde OC Krasnitz A Lundin P Naume B Sorlie T Borgen E Rye IH Langerod A Genomic architecture characterizes tumor progression paths and fate in breast cancer patients Sci Transl Med 2010 2 38ra47 10.1126/scitranslmed.3000611 PMC3972440 20592421

[B23] Stamps AC Davies SC Burman J O'Hare MJ Analysis of proviral integration in human mammary epithelial cell lines immortalized by retroviral infection with a temperature-sensitive SV40 T-antigen construct International journal of cancer Journal international du cancer 1994 57 865 874 10.1002/ijc.2910570616 8206680

[B24] Briand P Petersen OW van Deurs B A new diploid nontumorigenic human breast epithelial cell line isolated and propagated in chemically defined medium In vitro cellular & developmental biology: journal of the Tissue Culture Association 1987 23 181 188 10.1007/BF02623578 3558253

[B25] Pole JCM Courtay-Cahen C Garcia MJ Blood KA Cooke SL Alsop AE Tse DML Caldas C Edwards PAW High-resolution analysis of chromosome rearrangements on 8p in breast, colon and pancreatic cancer reveals a complex pattern of loss, gain and translocation Oncogene 2006 25 5693 5706 10.1038/sj.onc.1209570 16636668

[B26] Chua YL Ito Y Pole JC Newman S Chin SF Stein RC Ellis IO Caldas C O'Hare MJ Murrell A Edwards PA The NRG1 gene is frequently silenced by methylation in breast cancers and is a strong candidate for the 8p tumour suppressor gene Oncogene 2009 28 4041 4052 10.1038/onc.2009.259 19802002 PMC2789334

[B27] Bignell GR Greenman CD Davies H Butler AP Edkins S Andrews JM Buck G Chen L Beare D Latimer C Signatures of mutation and selection in the cancer genome Nature 2010 463 893 898 10.1038/nature08768 20164919 PMC3145113

[B28] Greenman CD Bignell G Butler A Edkins S Hinton J Beare D Swamy S Santarius T Chen L Widaa S PICNIC: an algorithm to predict absolute allelic copy number variation with microarray cancer data Biostatistics (Oxford, England) 2010 11 164 175 10.1093/biostatistics/kxp045 PMC2800165 19837654

[B29] Quail MA Swerdlow H Turner DJ Haines JL Improved protocols for the illumina genome analyzer sequencing system Current protocols in human genetics 2009 US: Wiley Chapter 18:Unit 18.12 10.1002/0471142905.hg1802s62 PMC3849550 19582764

[B30] Li H Durbin R Fast and accurate long-read alignment with Burrows-Wheeler transform Bioinformatics (Oxford, England) 2010 26 589 595 10.1093/bioinformatics/btp698 PMC2828108 20080505

[B31] Li H Handsaker B Wysoker A Fennell T Ruan J Homer N Marth G Abecasis G Durbin R Genome project data processing S: the sequence alignment/Map format and SAMtools Bioinformatics (Oxford, England) 2009 25 2078 2079 10.1093/bioinformatics/btp352 PMC2723002 19505943

[B32] Kent WJ BLAT–the BLAST-like alignment tool Genome Res 2002 12 656 664 11932250 10.1101/gr.229202 PMC187518

[B33] Conrad DF Bird C Blackburne B Lindsay S Mamanova L Lee C Turner DJ Hurles ME Mutation spectrum revealed by breakpoint sequencing of human germline CNVs Nat Genet 2010 42 385 391 10.1038/ng.564 20364136 PMC3428939

[B34] Greenman CD Pleasance ED Newman S Yang F Fu B Nik-Zainal S Jones D Lau KW Carter N Edwards PAW Estimation of rearrangement phylogeny for cancer genomes Genome Res 2012 22 346 361 10.1101/gr.118414.110 21994251 PMC3266042

[B35] Pole JCM McCaughan F Newman S Howarth KD Dear PH Edwards PAW Single-molecule analysis of genome rearrangements in cancer Nucleic Acids Res 2011 39 e85 10.1093/nar/gkr227 21525129 PMC3141271

[B36] Howarth KD Pole JC Beavis JC Batty EM Newman S Bignell GR Edwards PA Large duplications at reciprocal translocation breakpoints that might be the counterpart of large deletions and could arise from stalled replication bubbles Genome Research 2011 21 40 524 534 10.1101/gr.114116.110 PMC3065700 21252201

[B37] Bignell GR Santarius T Pole JCM Butler AP Perry J Pleasance E Greenman C Menzies A Taylor S Edkins S Architectures of somatic genomic rearrangement in human cancer amplicons at sequence-level resolution Genome Res 2007 17 1296 1303 10.1101/gr.6522707 17675364 PMC1950898

[B38] Hastings PJ Ira G Lupski JR A microhomology-mediated break-induced replication model for the origin of human copy number variation PLoS Genet 2009 5 e1000327 10.1371/journal.pgen.1000327 19180184 PMC2621351

[B39] Hicks J Krasnitz A Lakshmi B Navin NE Riggs M Leibu E Esposito D Alexander J Troge J Grubor V Novel patterns of genome rearrangement and their association with survival in breast cancer Genome Res 2006 16 1465 1479 10.1101/gr.5460106 17142309 PMC1665631

[B40] Hampton OA Koriabine M Miller CA Coarfa C Li J Den Hollander P Schoenherr C Carbone L Nefedov M Ten Hallers BF Long-range massively parallel mate pair sequencing detects distinct mutations and similar patterns of structural mutability in two breast cancer cell lines Cancer Genet 2011 204 447 457 10.1016/j.cancergen.2011.07.009 21962895 PMC3185296

[B41] Edwards PA Howarth KD Are breast cancers driven by fusion genes? Breast cancer research: BCR 2012 14 303 10.1186/bcr3122 22424054 PMC3446366

[B42] Fridlyand J Snijders AM Ylstra B Li H Olshen A Segraves R Dairkee S Tokuyasu T Ljung BM Jain AN Breast tumor copy number aberration phenotypes and genomic instability BMC Cancer 2006 6 96 10.1186/1471-2407-6-96 16620391 PMC1459181

[B43] Stratton MR Campbell PJ Futreal PA The cancer genome Nature 2009 458 719 724 10.1038/nature07943 19360079 PMC2821689

[B44] Zhao Q Caballero OL Levy S Stevenson BJ Iseli C de Souza SJ Galante PA Busam D Leversha MA Chadalavada K Transcriptome-guided characterization of genomic rearrangements in a breast cancer cell line Proc Natl Acad Sci USA 2009 106 1886 1891 10.1073/pnas.0812945106 19181860 PMC2633215

[B45] Banerji S Cibulskis K Rangel-Escareno C Brown KK Carter SL Frederick AM Lawrence MS Sivachenko AY Sougnez C Zou L Sequence analysis of mutations and translocations across breast cancer subtypes Nature 2012 486 405 409 10.1038/nature11154 22722202 PMC4148686

[B46] Ellis MJ Ding L Shen D Luo J Suman VJ Wallis JW Van Tine BA Hoog J Goiffon RJ Goldstein TC Whole-genome analysis informs breast cancer response to aromatase inhibition Nature 2012 486 353 360 22722193 10.1038/nature11143 PMC3383766

[B47] Curtis C Shah SP Chin SF Turashvili G Rueda OM Dunning MJ Speed D Lynch AG Samarajiwa S Yuan Y The genomic and transcriptomic architecture of 2,000 breast tumours reveals novel subgroups Nature 2012 486 346 352 22522925 10.1038/nature10983 PMC3440846

