# Supplementary material for: Structural analysis of the genome of breast cancer cell line ZR-75-30 identifies twelve expressed fusion genes
Source: BMC Genomics. 2012 Dec 22;13:719. doi: 10.1186/1471-2164-13-719 (PMC3548764; doi:10.1186/1471-2164-13-719)
Supplement: Additional file 1 — Junction and fusion transcript sequences. [file 1471-2164-13-719-S1.doc]

**Additional file 1:** Junction and fusion transcript sequences.

Exon numbers were taken from the following ensembl transcripts:

| **Name** | **Transcript ID** |
| --- | --- |
| COL14A1-001 | ENST00000297848 |
| SKAP1-201 | ENST00000336915 |
| APPBP2-201 | ENST00000083182 |
| PHF20L1-002 | ENST00000337920 |
| TAOK1-201 | ENST00000261716 |
| PCGF2-201 | ENST00000360797 |
| USP32-201 | ENST00000300896 |
| CCDC49 (CWC25-201) | ENST00000225428 |
| BCAS3-203 | ENST00000390652 |
| HOXB9-001 | ENST00000311177 |
| TIAM1-002 | ENST00000469412 |
| NRIP1-201 | ENST00000318948 |
| ZMYM4-001 | ENST00000314607 |
| OPRD1-001 | ENST00000234961 |
| TIMM23-001 | ENST00000260867 |
| ARHGAP32-001 | ENST00000310343 |
| TRPS1-001 | ENST00000395715 |
| LASP1-001 | ENST00000318008 |
| DDX5-203 | ENST00000540698 |
| DEPDC6-001 | ENST00000286234 |
| PLEC1-002 | ENST00000436759 |
| ENPP2-003 | ENST00000259486 |
| ERBB2-201 | ENST00000269571 |

Sequences are from cloned junctions (with the exception of DDX5-DEPDC6). Upper and lower case distinguish different fusion partners. Exons are separated by slash (/). Additional expressed chimeric transcripts are indicated where applicable.

**COL14A1-SKAP1**

Transcript 1 (in frame; COL14A1 exon 2 joined to SKAP1 exon 5):

...GCTACACCCCATGTAAAAAGCGGAAAATAAAATGAAGATTTTCCAGCGCAAGATGCGGTACTGGTTGCTTCCACCTTTTTTGGCAATTGTTTATTTCTGCACCATTGTCCAAGGTCAAGgaatggaagacatcgtaaaaggagctcaagaacttgataacgtaatcaagcaaggatacttggagaagaaaagcaaag...

Start codon (underlined) within COL14A1 exon 2

Transcript 2 (out of frame; fusion transcript including two cryptic exons from within SKAP1 intron 4 between COL14A1-exon 2 and SKAP1 exon 5):

...AGATTTTCCAGCGCAAGATGCGGTACTGGTTGCTTCCACCTTTTTTGGCAATTGTTTATTTCTGCACCATTGTCCAAGGTCAAG*acaattgccatgaaaaccctggcccatccccctacgtgcacggctatatgcaccaaagaagaatctaatatgcaacttcgtttccccattttaatccag*/*ttctctgcagatctgttcgtttaccagtaggtgtggatccaggcatacaagattcctgcctcatcaccccatggcatggctaacactgttcatcctcaggatcactagttcttgatcagtaggaccctgcgctgacactgaaagaggag*/gaatggaagacatcgtaaaaggagctcaagaacttgataacgtaatcaagcaaggatacttggagaagaaaagcaaag...

Transcript 3 (out of frame; fusion transcript incl. two cryptic exons from within SKAP1 intron 4 between COL14A1 exon 2 and SKAP1 exon 5, and lacking SKAP1 exon 7):

...AGATTTTCCAGCGCAAGATGCGGTACTGGTTGCTTCCACCTTTTTTGGCAATTGTTTATTTCTGCACCATTGTCCAAGGTCAAG*acaattgccatgaaaaccctggcccatccccctacgtgcgcggctatatgcaccaaagaagaatctaatatgcaacttcgtttccccattttaatccag*/*taggtgtggatccaggcatacaagattcctgcctcatcaccccatggcatggctaacactgttcatcctcaggatcactagttcttgatcagtaggaccctgcgctgacactgaaagaggag*/gaatggaagacatcgtaaaaggagctcaagaacttgataacgtaatcaagcaaggatacttggagaagaaaagcaaag/atcatagtttctttggatcggagtggcagaagcgatggtgtgttgtcagcagaggtctcttctactactatgctaatgagaaga/tttacagctactagtccagcagaagccagagactgggtggatcaaataagtttcttgttaaagg/atctgagctccttaaccattccatatgaagaggatgaggaggaagaagaaaaagaagagacatatgatgatattgatggttttgactccccaagttgtggttcccagtgcagacccactatcttgcctgggagtgtggggataaaagagcctacagaggagaaagaagaagaagatatttatgaagtcttgccag/atgaagagcatgatctagaagaggatgagagtggcactcgacgaaaaggag/tagactatgccagttactaccagggcctatgggattgccatggtgaccagccagatgaactgtccttccaacggggtgacctcatccgtattctgagcaag/gagtataacatgtatggctggtgggtgggagaactgaacagcctcgttgggattgttccaaagga...

**APPBP2-PHF20L1**

Transcript 1 (in frame; full-length product: APPBP2 exons 5-9 joined to PHF20L1 exons 3-8):

GCTCAGACATATATGGATAAACTATCAAAACATGGCCAGCAAGCAAATAAAGCTGCACTCTATGGAGAACTGTGTGCACTCCTATTTGCAAAAAGTCACTATGATGAG/GCATACAAATGGTGCATCGAGGCAATGAAAGAAATTACAGCAGGCTTACCAGTGAAAGTTGTGGTGGATGTCTTAAGACAAGCTTCTAAG/GCTTGTGTAGTAAAACGTGAATTTAAGAAGGCAGAACAGTTAATTAAACATGCAGTGTATTTGGCACG/GGATCATTTTGGATCCAAACACCCAAAATATTCTGATACACTGCTAGATTATGGGTTCTACTTACTCAATGTAGATAATATCTGTCAGTCTGTTGCAATTTATCAG/GCAGCCCTTGACATTAGACAGTCAGTGTTTGGTGGCAAAAATATCCACGTAGCAACAGCTCATGAAGATTTGGCCTACTCTTCTTATGTCCACCAGTATAGCTCTGGGAAATTTGACAATGCACTgtatccatcacgaattgaaaaaattgactatgaggagggcaagatgttggtccattttgagcgctggagtcatcgttatgatgagtggatttactgggatagcaatagattgcgaccccttgagagaccagcactaagaaaagaagggctaaaagatgaggaagatttcttt/gattttaaagctggagaagaagttctggctcgttggacagactgtcgctattaccctgccaagattgaagcaattaacaaagaag/gaacatttacagttcagttttatgatggagtaattcgttgtttaaaaagaatgcacattaaagccatgcccgaggatgctaaggggcag/gattggatagctttagtcaaagcagctgctgcagctgcagccaagaacaaaacagggagtaaacctcgaaccagcgctaacagcaataaagataaggataaagatgagagaaagtggtttaaagtaccttcaaagaaggaggaaacttcaacttgtatagccacaccagacgtagagaagaaggaagatctgcctacatctagtgaaacatttg/gacttcatgtagagaacgttccaaagatggtctttccacagccagagagcacattatcaaacaagaggaaaaataatcaaggcaactcgtttcaggcaaagagagctcgacttaacaagattactg/gtttgttggcatccaaagctgttggggttgatggtgctgaaaaaaaggaagactacaatgaaacagctccaatgctggagcaggtatgaaatggtagcatttgatttttttcaaggttcccactggaatgatca

Start codon (underlined) within APPBP2 exon 5 and stop codon after PHF20L1 exon 8

Transcript 2 (out of frame; APPBP2 exon 8 joined to PHF20L1 exon 3):

...GGATCATTTTGGATCCAAACACCCAAAATATTCTGATACACTGCTAGATTATGGGTTCTACTTACTCAATGTAGATAATATCTGTCAGTCTGTTGCAATTTATCAGgtatccatcacgaattgaaaaaattgactatgaggagggcaagatgttggtccattttgagcgctggagtcatcgttatgatgagtggatttactgggatagcaatagattgcgaccccttgagagaccagcactaagaaaagaagggctaaaagatgaggaagatttcttt...

**TAOK1-PCGF2**

Transcript (in frame; TAOK1 exon 1 joined to PCGF2 exon 3):

...CCTCACTCCTCACCCTCCAGGGTAGCGGCTA*CCGGA*atcatgcatcggactacacggatcaaaatcacagagctgaacccccacctcatgtgtgccctctgcggggggtacttcatcgacgccaccactatcgtggagtgcctgcattcct...

Start codons (underlined) within PCGF2 exon 3

*CCGGA*: is present in both the TAOK1 and the PCGF2 sequence

**USP32-CCDC49**

Transcript (out of frame; USP32 exon 2 joined to CCDC49 exon 3):

...TTACAGATGTAGAGCTAAAACGACTGAAGGATGCTTTCAAGAGGACCTGTGGACTCTCATATTACATGGGCCAGCACTGCTTCATCCGGGAAGTGCTTGGGGATGGAGTGCCTCCAAAGGTTGCTGAGgaaaaaaagaagaaaagttggactggatgtaccagggtcctggtgggatggtgaaccgtgacgagtacctgctggggcgccccattgacaaatatgtttttgagaagatggaggagaaggaggcaggctgctcttctgaaacaggacttctcccaggctctatctttgccccatcaggtgccaattcccttcttgacatggccagcaagatccgggaggacccactcttcatcatcag...

**BCAS3-HOXB9**

Transcript (out of frame; BCAS3 exon 7 joined to HOXB9 exon 2):

...GTGCTCAAAAATGTGATAACTTTGCTGAAAAAAGACCCCTCCTTGGTGTTTGTAAGAGCATTGGATCTTCTGGccaacccctccgccaactggctgcacgctcgctcttcccggaaaaagcgctgtccctacaccaaataccagacgctggagctagagaaggagtttctgttcaatatgtacctcaccagggaccgtaggcacgaagtggccagactcct...

**TIAM1-NRIP1**

Transcript (in frame; TIAM1 exon 1 joined to NRIP1 exon 2):

...GCCCGAGTCACTTACTCTGCCCAGCAGgaagtgtttggattgtgagctatttcagaactgttctcaggactcattattttaacatttgggagaaacacagccagaag...

**ZMYM4-OPRD1**

Transcript 1 (out of frame; ZMYM4 exons 25-26 joined to OPRD1 exon 2):

...GGGTTGAACAGGCCTCATCTAGCCCACGTTCTGACCCCTTAGGAAGTACTCAAGACCATGCACTCTCTCAAGAATCCTCAGAGCCAGGCTGTAGAG/TCCGCTCTATCAAGCTGAAGGAAGACATTCTGTCCTGCACTTTTGCTGAGTTGAGTTTGGGCTTATGCCAGTTTATCCAAGAGGTGCGGAGACCAAATGGTGAAAAATATGATCCAGACAGTATCTTATACTTGTGCCTTGGAATTCAACAGgtacactaagatgaagacggccaccaacatctacatcttcaacctggccttagccgatgcgctggccaccagcacgctgcctttccagagtgccaagtacctgatggagacgtggcccttcggcgagctgctctgcaaggctgtgctctccatcgacta...

Transcript 2 (out of frame; ZMYM4 exon 25 joined to OPRD1 exon 2):

...GGGTTGAACAGGCCTCATCTAGCCCACGTTCTGACCCCTTAGGAAGTACTCAAGACCATGCACTCTCTCAAGAATCCTCAGAGCCAGGCTGTAGAGgtacactaagatgaagacggccaccaacatctacatcttcaacctggccttagccgatgcgctggccaccagcacgctgcctttccagagtgccaagtacctgatggagacgtggcccttcggcgagctgctctgcaaggctgtgctctccatcgacta...

**TIMM23-ARHGAP32**

Transcript (out of frame: TIMM23 exon 6 joined to ARHGAP32 exon 2):

...ggtcatcattgagaaaacacgaggtgcagaagatgaccttaacacagtagcagctggaaccatgacaggcatgttgtataaatgtac*AGG*AAGATGAAGTCCTCAGTACATTCTGAAGAAGATGATTTTGTTCCAGAGCTACATAGAAATGTACACCCTCGAGAGCGGCCTGATTGGGAAGAAACTCTTAGCGCAAT...

**TRPS1-LASP1**

Transcript (in frame: TRPS1 exon 3 joined to LASP1 exon 2):

...AGCAAAATCTTGGCCCTTCATAACATGGTGCAGTTCAGCCATTCCAAAGACTTCCAGAAGGTCAACCGTTCTGTGTTTTCTGGTGTGCTGCAGGACATCAATTCTTCAAGGCCTGTTTTACTAAATGGGACCTATGATGTG*CAG*ttctggcataaagcatgcttccattgcgagacctgcaagatgacactgaacatgaagaactacaagggctacgagaagangccctactgcaacgcacactaccccaagcagtcctncaccatggtggcggacaccc...

**DDX5-DEPDC6**

Transcript (in frame: DDX5 exon 1 joined to DEPDC6 exon 2):

AATTGCCGTCTCTCTGTCAGAAGCCTCTTTGTGTTCAATCAGCCAGTCAATCAGTTCTTTTGCGACAAAACAGTTTGGGTAGGTCTTGAGATGATGACGTCTATCTTTAATAACCTTTTCTTCGTGCAGCCTGAGCcctcggtcccggcc

Gcggtctcggtanctcgaataaaannggtnac

**PLEC1-ENPP2**

Transcript (in frame: PLEC1 exon 2 joined to ENPP2 exon 4):

...tggccagcagtcctctgagcagtgacaggcattttcttcatttctgacttctccacatctgtccttagtacactcccagccacggg*CT*GCGATGCGAATGACCGCCCGCTCAGCTGGGTCCAGGACACTCCCGTTGCTCCCAGCGCCACCCCCGCTGCGCCGGCTCCGCTGCGTTTTCCCAAGGTTCCAGGGCAGTGTGT...

**ERBB2-BCAS3**

Transcript (out of frame: ERBB2 exon 19 joined to BCAS3 exon 2):

TACAAGGGCATCTGGATCCCTGATGGGGAGAATGTGAAAATTCCAGTGGCCATCAAAGTGTTGAGGGAAA ACACATCCCCCAAAGCCAACAAAGAAATCTTAGAC*GT*tttatgaatgaagctatggctacagattcccca agaagacccagtcgttgtactggtggagttgtggttcgcccccaggctgtcacagagcagtcctacatgg aaagtgttgtgaaagggcgaattcncggccgctaaattca
