# Supplementary material for: Structural analysis of the genome of breast cancer cell line ZR-75-30 identifies twelve expressed fusion genes
Source: BMC Genomics. 2012 Dec 22;13:719. doi: 10.1186/1471-2164-13-719 (PMC3548764; doi:10.1186/1471-2164-13-719)
Supplement: Additional file 3 — Genomic junction sequences. [file 1471-2164-13-719-S3.doc]

**Additional file 3:** Genomic junction sequences.

Upper and lower case letters are used to distinguish different fusion partners. Chromosome positions are based on the UCSC Genome Browser, hg19, GRCh build 37. Sequences common to both genes are shown in red.

**COL14A1-SKAP1**

ACTTCTTAATATGAATCATCTTTCAAAGAAACAGTGCAcacacatgaaataaagttttatattttgtatcttctc

chr17:46335633-46335673

chr8:121168715-121168752

**Chromosome 8-APPBP2**

GAGTCTTGTAGTAGATTACACGAGCAAAAGAAAGTCCGtttattttcattatgcagctctcccctttcaagctat

chr8:109678769-109678808

chr17:58546719-58546755

**APPBP2-PHF20L1**

ttagccgggcatggtggtgggcacttgtaatcccagctAACCTCCGCTCGCTGCAACCTCCGCCTCTCAGGTTCA

chr17:58536982-58537019 -strand

chr8:133793738-133793774 +strand

**TAOK1-PCGF2**

ATATGACCTTATACTAATTATTCTTAGCAGTTTTTGCCtgggctcagggagagatgtgctggtggggggaggtgt

chr17:27778403-27778441 +strand

chr17:36897070-36897106 –strand

**USP32-CCDC49**

TTATTTAGAAGTTTGGTGATATTTTTGTGACCAGAAACATACCTCAGGAA*actcctgagttcaggtgattctcctgcctcagcctcccgagtagctgggattacaggcatgtgccaccatgcctggctaatttttggttttttttttttgagacggagtctcgctctgtcgccaggctggagtacactggcgcaatctcagctcactgcaacctccaccttctgggttcaagcgattctcc*gcctctaagtaaataaataaataaataaataagtaaaaattgattttagactgaatacc

chr17:58397593-58397642 –strand

*chr17:36975540-36975730 +strand*

chr17:36975241-36975299 –strand

**BCAS3-HOXB9**

TCAGTGAGATGTAAGGAATCCTTGTCTGCTACTGGGCTtggcatttagggaacacattcttccccaaccctcccc

chr17:58905961-58905998 +strand

chr17:46701813-46701849 –strand

**TIAM1-NRIP1**

TTCAGTGACCACATCTTGGTATTTTAATTTTCTTTCTATgtgatattgaggcagattgattcatcatcatgaaga

chr21:16422419-16422457 +strand

chr21:32839194-32839230 +strand
