# Supplementary material for: Structural analysis of the genome of breast cancer cell line ZR-75-30 identifies twelve expressed fusion genes
Source: BMC Genomics. 2012 Dec 22;13:719. doi: 10.1186/1471-2164-13-719 (PMC3548764; doi:10.1186/1471-2164-13-719)
Supplement: Additional file 4 — Primers for amplifying genomic or transcript junctions and full-length fusion genes. [file 1471-2164-13-719-S4.doc]

**Additional file 4: Primers for amplifying genomic or transcript junctions and full-length fusion genes.**

| **Junction^a^** | **Breakpoint/junction** | **Primer name** | **Primer sequence (5’-3’)** |
| --- | --- | --- | --- |
| COL14A1-SKAP1 | Genomic junction | gCOL_F | GATCTCAGCTCCCTGCAGTC |
|  |  | gSKAP_F | GCATGCAAGAGGTTGATTTG |
|  | Transcript junction | COL14A1_2F | AGATTTTCCAGCGCAAGATG |
|  |  | SKAP1_5.6R | TGACAACACACCATCGCTTC |
|  | Full-length product | COL_FL | GCTACACCCCATGTAAAAAGC |
|  |  | SKAP1_FL | TGGGTTTCATCTTTCTTCCAC |
| Chr8-APPBP2 | Genomic junction | g8.1_R | CCAAGAGATGGGATGTGACC |
|  |  | gA1_F | TCAGCCAGACATGACGAAAG |
| APPBP2-PHF20L1 | Genomic junction | gAPPBP_R | AGCAAAGTCAAGACTGCAACC |
|  |  | gPHF_R | AAACAGCCCACACTACTCAGG |
|  | Transcript junction | APPBP2_7.8F | AACGTGAATTTAAGAAGGCAGAAC |
|  |  | PHF20L1_2.3R | ACCAACATCTTGCCCTCCTC |
|  |  | APPBP2_9.10F | TGGGAAATTTGACAATGCAC |
|  |  | PHF20L1_4.5R | CAACGAATTACTCCATCATAAAACTG |
|  | Full-length product | APPBP2_5F | GCTCAGACATATATGGATAAACTATCA |
|  |  | PHF20L1_8UTR | TGATCATTCCAGTGGGAACC |
| USP32-CCDC49 | Genomic junction | gUSP_R | GTGCACGTTTGATGACTTGG |
|  |  | gCCDC_F | ACAATGCTGGCTCCTTTCTC |
|  | Transcript junction | USP32_1.2F | TCGGATTCCTCAGCTACGAG |
|  |  | CCDC49_3.4R | TTCACTGGATTATTTAATACCTCTCG |
| TAOK1-PCGF2 | Genomic junction | gTAOK1_F | GAGATGACTGCATCAGAAGTGG |
|  |  | gPCGF2_F | GGAAACGGAAGTGGAATCAG |
|  | Transcript junction | TAOK1_1.2F | CCTCACTCCTCACCCTCCAG |
|  |  | PCGF2_4.5R | CCCAGGGACCAATTTGTAGAC |
| BCAS3-HOXB9 | Genomic junction | gBCAS3_F | TTCCATACAGGCATGCTTACTC |
|  |  | gHOXB9_F | CCCCAAGTGAAAAACACTGG |
|  | Transcript junction | BCAS3_1.2F | CCAAGAAGACCCAGTCGTTG |
|  |  | HOXB9_2R | CACTCAGATTGAGGAGTCTGG |
|  | Full-length product | BCAS3_5UTR | CAGTGAGCCTAGAGCTGGAGA |
|  |  | HOXB9_3UTR | GAGATGGGGAAGAGCTAGGG |
| TIAM1-NRIP1 | Genomic junction | gTIAM1_R | ATTTGGGGATTATGGAACTTTG |
|  |  | gNRIP1_F | CAAGCCAATCTTTTGCAACTTAC |
|  | Transcript junction | TIAM1_1F | ACCCTGCCCGAGTCACTTAC |
|  |  | NRIP1_1R | CTTTGACAGGTGGGAAATGC |
| ZMYM4-OPRD1 | Genomic junction | gZMYM_four_F | AGGATTCTGTCACCTGCAAC |
|  |  | gOPRD_one_R | AGCATTTTGGGTGCCAAG |
|  | Transcript junction | ZMYM4_22F | ACTGAACAGGACCTGGAAGC |
|  |  | OPRD1_2R | TAGTCGATGGAGAGCACAGC |
| TIMM23-ARHGAP32 | Genomic junction | BP15F | TATGGAGGAAGAAGTCCATGA |
|  |  | BP15R | CGTTGCAGAAATGGTTAGTTC |
|  | Transcript junction | TIMM23ex5F | GGCACTTTGGGCTAATACTCT |
|  |  | ARHGAP32ex2R | ATTGCGCTAAGAGTTTCTTCC |
| TRPS1-LASP1 | Genomic junction | BP44F | GGTTGGATGCTGTAGAGGAGT |
|  |  | BP44R | AGTTCTTTCATCAAGCCAGGT |
|  | Transcript junction | TRPS1ex2F | ATGAAGTCAATGCTGGGTATG |
|  |  | LASP1ex3R | GCTTGAGGCGAAGGTTTT |
| DDX5-DEPDC6 | Transcript junction | DDX5ex1F | TTATTCGAGTGACCGAGACC |
|  |  | DEPDC6ex3R | GGCCTTCACTTCATTATCCAA |
| PLEC1-ENPP2 | Transcript junction | PLEC1ex2F | ACACACTGCCCTGGAACCT |
|  |  | ENPP2ex5R | CACAGTCATCATCAACCCAAT |
| ERBB2-BCAS3 | Transcript junction | ERBB2ex14F | CCCCAGGGAGTATGTGAATG |
|  |  | BCAS3ex3bR | TCACAACACTTTCCATGTAGGAC |

^a^ Junction sequences are given in Additional files 1 and 3.
