# Supplementary material for: Structural analysis of the genome of breast cancer cell line ZR-75-30 identifies twelve expressed fusion genes
Source: BMC Genomics. 2012 Dec 22;13:719. doi: 10.1186/1471-2164-13-719 (PMC3548764; doi:10.1186/1471-2164-13-719)
Supplement: Additional file 6 — A comparison of breakpoints by 1Mb array painting and solexa sequencing data. [file 1471-2164-13-719-S6.pdf]

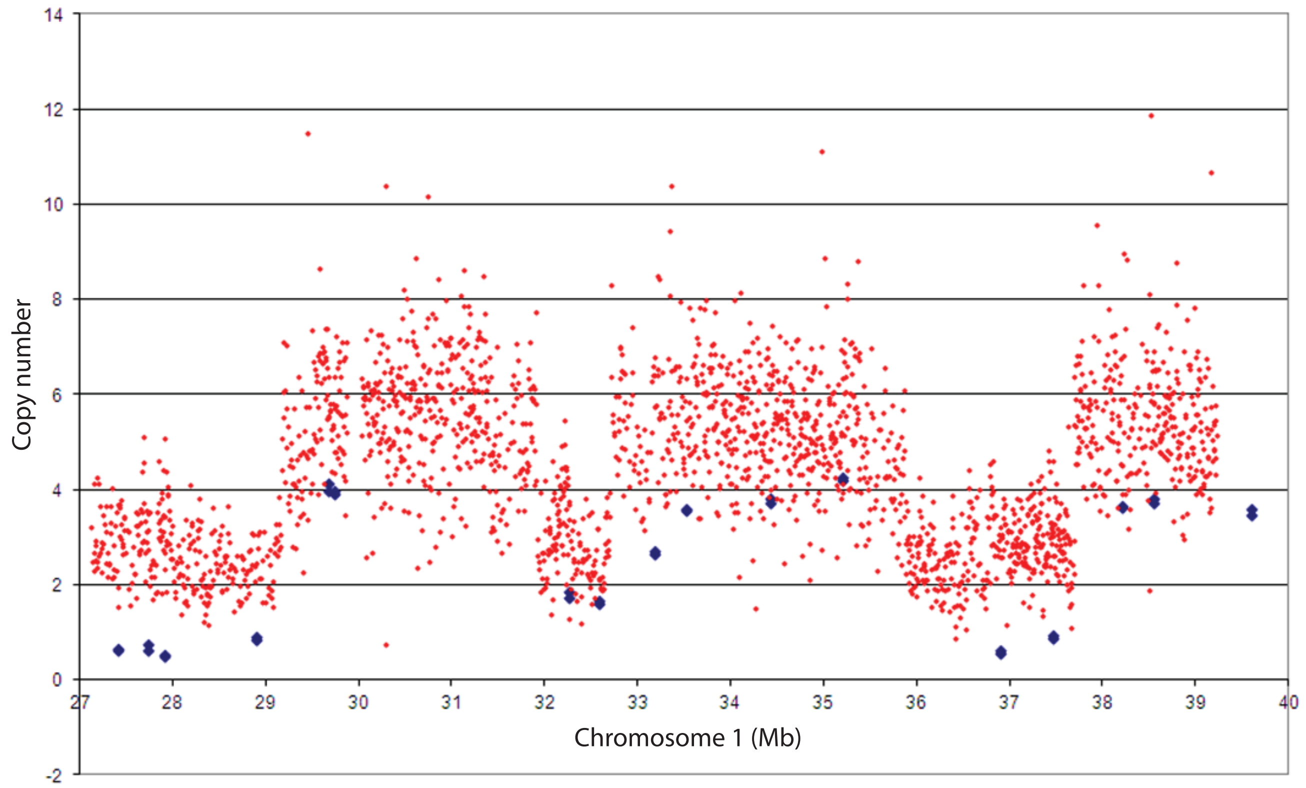

Additional file 6: A comparison of breakpoints by 1Mb array painting and solexa sequencing data.

Hybridisation of flow-sorted chromosome peak G (Howarth et al 2008) (blue diamonds) to a 1Mb BAC array is shown compared to copy number data from solexa sequencing (red diamonds). Copy number data is shown for the region spanning 27-40 Mb on chromosome 1 (Hg19). The pattern observed is strikingly similar and allows breakpoints to be refined to around 70kb resolution.
