# Supplementary material for: Structural analysis of the genome of breast cancer cell line ZR-75-30 identifies twelve expressed fusion genes
Source: BMC Genomics. 2012 Dec 22;13:719. doi: 10.1186/1471-2164-13-719 (PMC3548764; doi:10.1186/1471-2164-13-719)
Supplement: Additional file 8 — One possible assembly of ten junctions in the 8;17 amplicon of ZR-75-30. [file 1471-2164-13-719-S8.pdf]

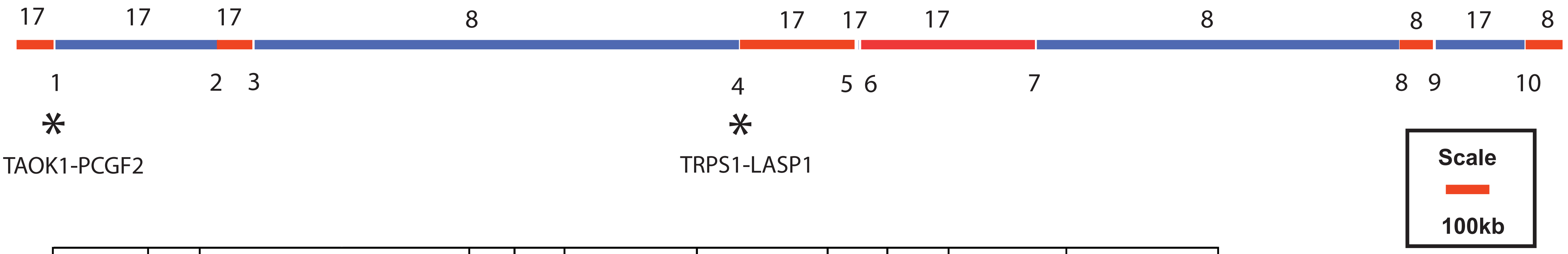

		Solexa reads (Hg19)									
**Junction**	**Chr**	**Node 1 start**	**node 1 end**	**Dir**	**Chr**	**Node 2 start**	**Node 2 end**	**Dir**	**SV**	**Supp reads**	**Confirmation**
**1**	17	36896659	36897109	+	17	27777987	27778098	+	INV	8	PCR
**2**	17	27403809	27404233	-	17	47560594	47561036	+	INS	22	snp6
**3**	17	47480427	47480775	-	8	117735269	117735665	+	DIF	16	snp6
**4**	8	116623415	116623774	-	17	37016564	37016943	-	DIF	7	PCR
**5**	17	37280837	37281318	+	17	28100421	28101123	-	INS	23	PCR+snp6
**6**	17	28100946	28101124	+	17	35142597	35142819	-	DEL	13	snp6
**7**	17	35544634	35545063	+	8	109665984	109666458	-	DIF	12	PCR+snp6
**8**	8	110498209	110498651	+	8	102603779	102604269	+	INV	66	PCR+snp6
**9**	8	102526951	102527414	-	17	26664041	26664498	-	DIF	103	snp6
**10**	17	26868545	26868937	+	8	143340183	143340539	+	DIF	23	snp6

Additional file 8: One possible assembly of ten junctions in the 8;17 amplicon of ZR7530. Segments of chromosmes 8 and 17 are shown to scale, in alternating red and blue for ease of visualisation. Two expressed, in-frame fusions (marked *) are shown. Junction numbers 1-10 are marked and the corresponding paired-end reads (Hg19) are shown in the table. All individual junctions were confirmed by genomic PCR or matching to a snp6 copy number step.
