# Supplementary material for: Structural analysis of the genome of breast cancer cell line ZR-75-30 identifies twelve expressed fusion genes
Source: BMC Genomics. 2012 Dec 22;13:719. doi: 10.1186/1471-2164-13-719 (PMC3548764; doi:10.1186/1471-2164-13-719)
Supplement: Additional file 9 — Supplementary discussion: Discussion of individual fusion genes. [file 1471-2164-13-719-S9.doc]

# Supplementary Discussion

## Individual fusion genes in the amplicon

### APPBP2-PHF20L1

This fusion transcript is probably incomplete, with additional unknown sequences 5’ to those cloned, i.e. it is geneX-APPBP2-PHF20L1.

The APPBP2 (amyloid protein-binding protein 2) fragment, amino acids 193-353, includes functional domains—at least 2 of the protein’s 4 tetratrico peptide repeat (TPR) domains and a domain similar to the kinesin light chain. APPBP2 is associated with PAT1/ARA67 protein [1], a modulator of the androgen receptor [2], and the androgen receptor may play a role in estrogen-independent breast cancers [3].

PHF20L1 (PHD finger protein 20-like 1) is related to chromatin-modifier proteins that are fused in other neoplasms. It has several isoforms, of which only isoform 2 participates in the fusion. This isoform has two Tudor domains, but does not include the plant homeodomain (PHD) zinc-finger domain present in other isoforms. The fusion includes exons 3 to 8, almost all of PHF20L1 isoform 2 (exons 1-8), including part of the first Tudor domain and all of the second.

Similar coding sequences, exons 1-8, are fused in the breast cancer cell line HCC1954 [4]. Because this fusion, to SAMD12, is out of frame, it encodes essentially the same protein as the normal isoform 2. Other PHF genes form fusion genes— including JAZF1-PHF1 and EPC1-PHF1 in endometrial stromal sarcomas, and NUP98-PHF23 in acute myeloid leukaemia [5]—and these also preserve the Tudor domains.

### BCAS3-HOXB9

This fusion would seem particularly likely to be functionally significant, because BCAS3 is fused in other cases, and fusions of other HOX genes are important in leukaemias.

BCAS3 (breast carcinoma amplified sequence 3) encodes three WD repeats. It is reported to be regulated by estrogen receptor alpha (ER-α) and may be a coactivator of ER-α, possibly amplifying ER-α signalling by positive feedback [6]; it may also regulate cell migration [7]. The fusion includes exons 1 to 7.

BCAS3 has also been found fused in a breast tumour [8], and in breast cancer cell lines MCF7 [9-10], BT474 and EFM-19 [11-12], and broken in HCC1806 and SUM52 ([13] and data not shown).The present fusion and the fusion in MCF7 are highly amplified, suggesting functional activity. However, the fusions retain different fragments of the gene—for example, the BCAS4-BCAS3 fusion in MCF7 retains only the two most 3’ exons of BCAS3, exons 23 and 24 [9]; while HCC1806 retains exon 6 onwards. The fusions may therefore be loss-of-function mutations for BCAS3, or, because BCAS3 is in a region often amplified in breast cancer, may represent random breakage of the gene.

HOXB9 is a homeobox gene. It is a critical player in development of mammary gland and is transcriptionally regulated by estrogen receptor [14]. Overexpression has been reported in breast cancers [15]. HOX genes form the 3’ gene of several gene fusions, including NUP98-HOXA9 and MSI2-HOXA9 in leukaemias, indeed, Look [16] suggested that many acute leukaemia translocations directly or indirectly targeted HOX activity. HOXA9 fusions are particularly important, and HOXA9 and HOXB9 are paralogues. The BCAS3-HOXB9 fusion retains the DNA-binding domain—the homeobox domain—but there is loss of the activation domain.

The fusion transcript that we detected, BCAS3 exon 7 - HOXB9 exon 2, retains the HOX domain of HOXB9, so, by analogy with the HOXA9 fusions, we would expect the protein to be translated. The published translation reading frame of BCAS3 [6, 9], which is highly conserved and encodes recognisable protein domains, joins HOXB9 out of frame. However, an alternative reading frame for BCAS3, starting at a methionine in exon 6, joins HOXB9 in frame to encode a predicted 138 amino-acid protein that includes the HOX domain of HOXB9 (Supplementary Table 4).

### COL14A1-SKAP1

The N terminus of the fusion protein is a signal peptide, the first 30 amino acids of collagen 14 alpha 1 (COL14A1), fused in frame to the signalling adaptor protein SKAP1 (src kinase family-associated phosphoprotein 1, also known as SKAP-55). The COL14A1 signal peptide presumably anchors the fusion protein to a membrane or directs its export from the cytoplasm. Thus, the fusion may alter the subcellular localisation of SKAP1, perhaps activating or stabilising it. Whether the collagen promoter also alters SKAP1 expression is not clear, as SKAP1 expression was found to vary greatly among breast cell lines, but this collagen is normally deposited in human mammary gland [17]. Several other fusion genes are known in solid tumours that include collagens as 5’ fusion partners, including COL1A1–PDGFB (collagen type 1 alpha 1 - platelet-derived growth factor beta) in dermatofibrosarcoma protuberans and giant cell fibroblastoma, and COL1A1–USP6 in aneurysmal bone cysts [18].

SKAP1 protein is a signalling adaptor without intrinsic enzymatic activity. In T cells it interacts with the MAPkinase and NFkappabeta pathways, and promotes the clustering of integrins on the cell surface [19]. The fusion preserves the C-terminal 265 amino acids, including the PH and SH3 domains, and the tyrosines that, when phosphorylated, bind to ADAP, FYN or RASGRP1 [20].

Unbalanced rearrangements within SKAP1 have also been found in the breast cancer cell lines BT474 [21] and HCC2218 [22], but in HCC2218 the 5’ end of the gene is retained, and the fusion in BT474 reported as ‘THRA-SKAP1’ [11] is on the opposite strand to SKAP1.

In conclusion, the structure of the fusion and its amplification suggests that it activates the signalling molecule SKAP1, but the contrasting structure of the other known rearrangements of SKAP1 might suggest that all the rearrangements may simply inactivate SKAP1 or be unrelated consequences of frequent rearrangement on 17q.

### TAOK1-PCGF2/MEL18

This fusion places the complete PCGF2 protein under the control of TAOK1 regulatory sequences, since it joins the untranslated TAOK1 exon 1 to the first translated exon of PCGF2, exon 3. PCGF2 (polycomb group RING finger protein 2, also known as MEL18, ZNF144, or RNF110) is a component of some forms of the chromatin-associated polycomb repressive complex 1 (PRC1) which maintains epigenetic repression of transcription, by compacting chromatin and monoubiquitylating histone H2A [23]. PCGF2 is a paralogue of BMI1, an alternative PRC1 component. BMI1 is amplified in B cell lymphoma and cooperates with Myc in a mouse model of lymphoma [24]. A possible fusion of PCGF2, that would retain all coding sequence, has been predicted from genomic paired-end sequencing data in a breast tumour [25] but it was not confirmed by PCR.

TAOK1 (thousand and one amino acid protein 1) is a serine/threonine kinase that activates the p38 MAP kinase pathway. A TAOK1-ELL fusion has been described in a breast tumour, which retains most of the coding sequence [25], while an out-of-frame PAX5-TAOK1 fusion, which encodes no TAOK1 protein, has been described in acute B lymphoblastic leukaemia (B-ALL) [26]. These fusions may each inactivate a copy of TAOK1.

This fusion may therefore have two activities: activation of a polycomb-group protein and inactivation of TAOK1.

### TRPS1-LASP1

The transcription factor TRPS1 is joined in frame to exon 2 of LASP1 (LIM and SH3 protein 1). The GATA-type zinc finger domain of TRPS1 is removed in the fusion and the LIM-type zinc finger domain of LASP1 is disrupted. However, the actin binding domains and SH3 domain of LASP1 are retained. LASP1 protein is a focal adhesion protein overexpressed in breast and ovarian cancer, and nuclear localisation of LASP1 correlates with poor survival in breast cancer patients [27]. An ARID5A-TRPS1 fusion has been described in a breast tumour [25], and an MLL-LASP1 and LASP1-MLL reciprocal fusion has been described in an acute leukaemia [28].

### USP32-CCD49

USP32-exon 2 is joined to CCDC49-exon 3 out of frame (Figure 2, Supplementary Table 2), so the transcript encodes little protein. USP32 (ubiquitin specific protease 32) is a membrane-anchored thiol protease involved in de-ubiquitination. CCDC49 (coiled-coil domain containing protein 49) is a phosphoprotein of unknown function. The rearrangement presumably inactivates a copy of each gene.

### Fusions identified by Robinson et al [12], DDX5-DEPDC6, ERBB2-BCAS3 and PLECC1-ENPP2

In both the DDX5-DEPDC6/DEPTOR and PLEC1-ENPP2 fusions the 5’ gene provides little more than a new promoter and initiating AUG, while the 3’ gene is almost intact (Figure 2C). DEPDC6/DEPTOR (DEP domain containing MTOR-interacting protein) inhibits the kinase activity of both the mTORC1 and mTORC2 signalling pathways. ENPP2 protein is reported to be upregulated in cancers and to stimulate cell motility (Refseq database, www.ncbi.nlm.nih.gov/RefSeq/).

The ERBB2-BCAS3 fusion is out of frame, with a STOP two amino acids after the junction, and is predicted to encode a truncated ERBB2 that includes the extracellular sequences, transmembrane domain and about 100 amino acids C-terminal of it, including roughly the first 50 of the 250 amino acid tyrosine kinase domain. Some ERBB2 transcripts, e.g. ENST00000540042, terminate 5’ to the fusion gene junction, so the rearranged gene may also express these shorter isoforms intact and unfused.

## Individual fusion genes not amplified

### TIAM1-NRIP1

This fusion could be relevant to estrogen receptor function—of interest in this cell line since it is estrogen-receptor-positive (ER+ve) but anti-estrogen resistant [29]. NRIP1 (nuclear receptor interacting protein 1/ receptor interacting protein 140, RIP140) is a nuclear phosphoprotein that complexes with agonist-liganded nuclear receptors including the estrogen receptor (ESR1) and androgen receptor [30]. There is also a breast cancer susceptibility locus within the gene [31]. The fusion places a normal NRIP1 protein under the control of the TIAM1 (cytosolic T-lymphoma invasion and metastasis-inducing protein 1) promoter, since exon 1 of TIAM1 is untranslated and the NRIP1 translation start site is downstream of the fusion junction. TIAM1 is widely expressed.

### TIMM23-ARHGAP32

This out-of-frame fusion of TIMM23 exon 6 to ARHGAP32 exon 2 would encode a truncated TIMM23 protein (TIMM23 has 7 exons). TIMM23 (translocase of inner mitochondrial membrane 23) is a component of the TIM complex, which facilitates the translocation of proteins across the inner mitochondrial membrane. The fusion would also inactivate a copy of ARHGAP32, Rho GTPase-activating protein 32, which is mutated in lung and ovarian cancers (COSMIC database, www.sanger.ac.uk/perl/genetics/CGP/cosmic).

### ZMYM4-OPRD1

This fusion presumably produces a truncated ZMYM4 protein and inactivates a copy of OPRD1. ZMYM4 (zinc finger MYM-type protein 4) contains nine MYM-type zinc fingers, which are retained in the fusion, and interacts with DNA. OPRD1 is opioid/enkephalin receptor delta 1, a G-protein coupled receptor.

# References

1. Zheng P, Eastman J, Vande Pol S, Pimplikar SW: **PAT1, a microtubule-interacting protein, recognizes the basolateral sorting signal of amyloid precursor protein.** *Proceedings of the National Academy of Sciences of the United States of America* 1998, **95:**14745-14750.

2. Hsu C-L, Chen Y-L, Ting H-J, Lin W-J, Yang Z, Zhang Y, Wang L, Wu C-T, Chang H-C, Yeh S, et al: **Androgen receptor (AR) NH2- and COOH-terminal interactions result in the differential influences on the AR-mediated transactivation and cell growth.** *Molecular endocrinology (Baltimore, Md)* 2005, **19:**350-361.

3. Robinson JLL, Macarthur S, Ross-Innes CS, Tilley WD, Neal DE, Mills IG, Carroll JS: **Androgen receptor driven transcription in molecular apocrine breast cancer is mediated by FoxA1.** *The EMBO journal* 2011, **30:**3019-3027.

4. Zhao Q, Caballero OL, Levy S, Stevenson BJ, Iseli C, de Souza SJ, Galante PA, Busam D, Leversha MA, Chadalavada K, et al: **Transcriptome-guided characterization of genomic rearrangements in a breast cancer cell line.** *Proceedings of the National Academy of Sciences of the United States of America* 2009, **106:**1886-1891.

5. Baker LA, Allis CD, Wang GG: **PHD fingers in human diseases: disorders arising from misinterpreting epigenetic marks.** *Mutation research* 2008, **647:**3-12.

6. Gururaj AE, Peng S, Vadlamudi RK, Kumar R: **Estrogen induces expression of BCAS3, a novel estrogen receptor-alpha coactivator, through proline-, glutamic acid-, and leucine-rich protein-1 (PELP1).** *Mol Endocrinol* 2007, **21:**1847-1860.

7. Jain M, Bhat GP, Vijayraghavan K, Inamdar MS: **Rudhira/BCAS3 is a cytoskeletal protein that controls Cdc42 activation and directional cell migration during angiogenesis.** *Experimental cell research* 2012, **318:**753-767.

8. Ellis MJ, Ding L, Shen D, Luo J, Suman VJ, Wallis JW, Van Tine BA, Hoog J, Goiffon RJ, Goldstein TC, et al: **Whole-genome analysis informs breast cancer response to aromatase inhibition.** *Nature* 2012, **486:**353-360.

9. Bärlund M, Monni O, Weaver JD, Kauraniemi P, Sauter G, Heiskanen M, Kallioniemi O-P, Kallioniemi A: **Cloning of BCAS3 (17q23) and BCAS4 (20q13) genes that undergo amplification, overexpression, and fusion in breast cancer.** *Genes, chromosomes & cancer* 2002, **35:**311-317.

10. Hampton OA, Den Hollander P, Miller CA, Delgado DA, Li J, Coarfa C, Harris RA, Richards S, Scherer SE, Muzny DM, et al: **A sequence-level map of chromosomal breakpoints in the MCF-7 breast cancer cell line yields insights into the evolution of a cancer genome.** *Genome Research* 2009, **19:**167-177.

11. Kim D, Salzberg SL: **TopHat-Fusion: an algorithm for discovery of novel fusion transcripts.** *Genome biology* 2011, **12:**R72.

12. Robinson DR, Kalyana-Sundaram S, Wu Y-M, Shankar S, Cao X, Ateeq B, Asangani IA, Iyer M, Maher CA, Grasso CS, et al: **Functionally recurrent rearrangements of the MAST kinase and Notch gene families in breast cancer.** *Nature medicine* 2011, **17:**1646-1651.

13. Howarth KD, Blood KA, Ng BL, Beavis JC, Chua Y, Cooke SL, Raby S, Ichimura K, Collins VP, Carter NP, Edwards PAW: **Array painting reveals a high frequency of balanced translocations in breast cancer cell lines that break in cancer-relevant genes.** *Oncogene* 2008, **27:**3345-3359.

14. Ansari KI, Shrestha B, Hussain I, Kasiri S, Mandal SS: **Histone methylases MLL1 and MLL3 coordinate with estrogen receptors in estrogen-mediated HOXB9 expression.** *Biochemistry* 2011, **50:**3517-3527.

15. Hayashida T, Takahashi F, Chiba N, Brachtel E, Takahashi M, Godin-Heymann N, Gross KW, Vivanco MdM, Wijendran V, Shioda T, et al: **HOXB9, a gene overexpressed in breast cancer, promotes tumorigenicity and lung metastasis.** *Proceedings of the National Academy of Sciences of the United States of America* 2010, **107:**1100-1105.

16. Look AT: **Oncogenic transcription factors in the human acute leukemias.** *Science (New York, NY)* 1997, **278:**1059-1064.

17. Atherton AJ, Warburton MJ, O'Hare MJ, Monaghan P, Schuppan D, Gusterson BA: **Differential expression of type XIV collagen/undulin by human mammary gland intralobular and interlobular fibroblasts.** *Cell and tissue research* 1998, **291:**507-511.

18. Oliveira AM, Perez-Atayde AR, Dal Cin P, Gebhardt MC, Chen C-J, Neff JR, Demetri GD, Rosenberg AE, Bridge JA, Fletcher JA: **Aneurysmal bone cyst variant translocations upregulate USP6 transcription by promoter swapping with the ZNF9, COL1A1, TRAP150, and OMD genes.** *Oncogene* 2005, **24:**3419-3426.

19. Raab M, Wang H, Lu Y, Smith X, Wu Z, Strebhardt K, Ladbury JE, Rudd CE: **T cell receptor "inside-out" pathway via signaling module SKAP1-RapL regulates T cell motility and interactions in lymph nodes.** *Immunity* 2010, **32:**541-556.

20. Raab M, Smith X, Matthess Y, Strebhardt K, Rudd CE: **SKAP1 protein PH domain determines RapL membrane localization and Rap1 protein complex formation for T cell receptor (TCR) activation of LFA-1.** *The Journal of biological chemistry* 2011, **286:**29663-29670.

21. Raphael BJ, Volik S, Yu P, Wu C, Huang G, Linardopoulou EV, Trask BJ, Waldman F, Costello J, Pienta KJ, et al: **A sequence-based survey of the complex structural organization of tumor genomes.** *Genome biology* 2008, **9:**R59.

22. Bignell GR, Greenman CD, Davies H, Butler AP, Edkins S, Andrews JM, Buck G, Chen L, Beare D, Latimer C, et al: **Signatures of mutation and selection in the cancer genome.** *Nature* 2010, **463:**893-898.

23. Margueron R, Reinberg D: **The Polycomb complex PRC2 and its mark in life.** *Nature* 2011, **469:**343-349.

24. Bracken AP, Helin K: **Polycomb group proteins: navigators of lineage pathways led astray in cancer.** *Nature reviews Cancer* 2009, **9:**773-784.

25. Banerji S, Cibulskis K, Rangel-Escareno C, Brown KK, Carter SL, Frederick AM, Lawrence MS, Sivachenko AY, Sougnez C, Zou L, et al: **Sequence analysis of mutations and translocations across breast cancer subtypes.** *Nature* 2012, **486:**405-409.

26. Coyaud E, Struski S, Prade N, Familiades J, Eichner R, Quelen C, Bousquet M, Mugneret F, Talmant P, Pages M-P, et al: **Wide diversity of PAX5 alterations in B-ALL: a Groupe Francophone de Cytogenetique Hematologique study.** *Blood* 2010, **115:**3089-3097.

27. Frietsch JJ, Grunewald TGP, Jasper S, Kammerer U, Herterich S, Kapp M, Honig A, Butt E: **Nuclear localisation of LASP-1 correlates with poor long-term survival in female breast cancer.** *British journal of cancer* 2010, **102:**1645-1653.

28. Strehl S, Borkhardt A, Slany R, Fuchs UE, König M, Haas OA: **The human LASP1 gene is fused to MLL in an acute myeloid leukemia with t(11;17)(q23;q21).** *Oncogene* 2003, **22:**157-160.

29. Engel LW, Young NA, Tralka TS, Lippman ME, O'Brien SJ, Joyce MJ: **Establishment and characterization of three new continuous cell lines derived from human breast carcinomas.** *Cancer research* 1978, **38:**3352-3364.

30. Fritah A, Christian M, Parker MG: **The metabolic coregulator RIP140: an update.** *American journal of physiology Endocrinology and metabolism* 2010, **299:**E335-340.

31. Ghoussaini M, Fletcher O, Michailidou K, Turnbull C, Schmidt MK, Dicks E, Dennis J, Wang Q, Humphreys MK, Luccarini C, et al: **Genome-wide association analysis identifies three new breast cancer susceptibility loci.** *Nature genetics* 2012, **44:**312-318.
